# A Toolbox for Diverse Oxyfunctionalisation of Monoterpenes

**DOI:** 10.1038/s41598-018-32816-1

**Published:** 2018-09-26

**Authors:** Aitor Hernandez-Ortega, Maria Vinaixa, Ziga Zebec, Eriko Takano, Nigel S. Scrutton

**Affiliations:** School of Chemistry, Manchester Institute of Biotechnology, University of Manchester, Manchester, M1 7DN United Kingdom

## Abstract

The successful implementation of synthetic biology for chemicals biosynthesis relies on the availability of large libraries of well-characterized enzymatic building blocks. Here we present a scalable pipeline that applies the methodology of synthetic biology itself to bootstrap the creation of such a library. By designing and building a cytochrome P450 enzyme collection and testing it in a custom-made untargeted GC/MS-metabolomics-based approach, we were able to rapidly create and characterize a comprehensive enzyme library for the controlled oxyfunctionalisation of terpene scaffolds with a wide range of activities and selectivities towards several monoterpenes. This novel resource can now be used to access the extensive chemical diversity of terpenoids by pathway engineering and the assembly of biocatalytic cascades to subsequently produce libraries of oxygenated terpenoids and their derivatives for diverse applications, including drug discovery.

## Introduction

Biosynthetic expansion of the chemical diversity of terpenes is a major challenge for biocatalysis and synthetic biology programmes in order to access new chemical space. Establishing a biocatalyst toolkit that performs site-selective oxyfunctionalisation of monoterpene hydrocarbons is required to make added-value products or to produce intermediates for subsequent tailoring by other enzymes. Oxyfunctionalisation of terpene scaffolds based on cytochrome P450 (CYP) dependent catalysis has been proposed as a route for expansion of chemical diversity, enabling the biosynthesis of high value-added end products^1,2^. CYPs offer several advantages as biocatalysts. They can, for example, oxidise inert C-H bonds and they have a broad substrate scope, regio- and stereo-selectivities^3,4^. Some CYPs were reported to be capable of oxygenating a few monoterpene scaffolds^2,5,6^. This foundational information was the starting point to develop a comprehensive CYP toolbox for the enzymatic tailoring of monoterpenes. Our CYP toolkit was carefully designed to overcome limiting factors (low protein solubility, cofactor incorporation, co-expression of redox partners) typically associated with CYPs^7^.

**Figure 1:**
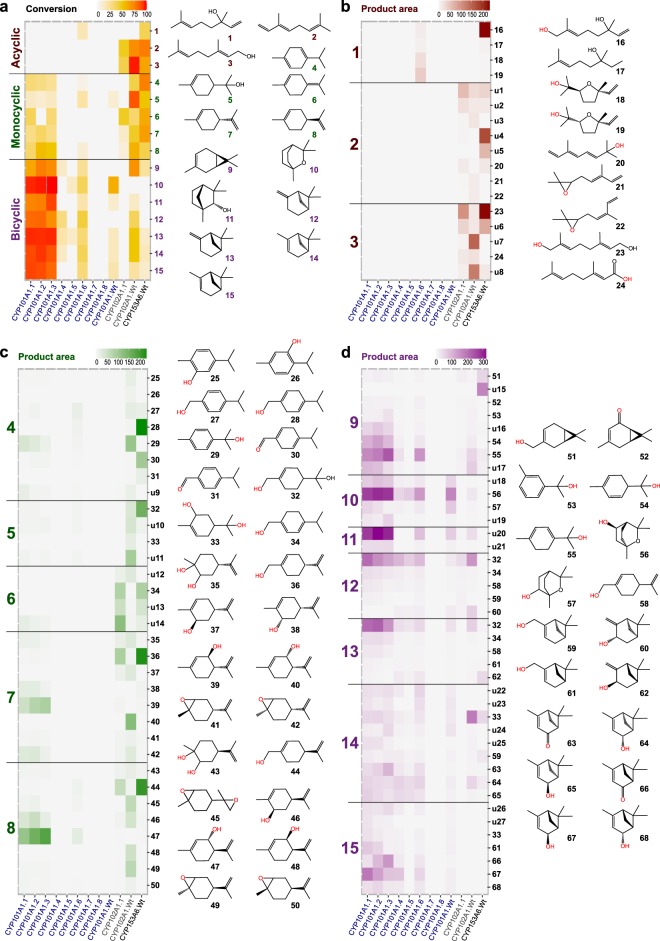
Untargeted GC/MS screening of the CYPs library. (**a**) Conversion of monoterpene substrates by the CYP library. Acyclic (brown), monocyclic (green) and bicyclic monoterpenes (lilac). (**b**–**d**) Product landscapes from biotransformation of acyclic, monocyclic and bicyclic substrates, respectively. Product conversions shown are calculated as described in the methods section. Structures of identified products are shown. Mass spectra of unidentified products are described in the online Supplementary Table S16.

Reactivities and selectivities for each member in our CYP toolbox were assayed across 15 different substrates representative of acyclic ((+/−)-linalool, (*E*/*Z*)-β-ocimene, geraniol; **1**–**3**), monocyclic (γ-terpinene, α-terpienol, terpinolene, (*R*)- and (*S*)-limonene; **4**–**8**) and bicyclic ((+)-carene, 1,8-cineole, (+)-fenchol, (−)- and (+)-β-pinene, (−)- and (+)-α-pinene; **9**–**15**) monoterpene scaffolds (Fig. 1). All enzymes were tested using a cell-free extract in the presence of high substrate concentrations and a glucose dehydrogenase NADH/NADPH recycling system to ensure multiple turnover conditions (Methods Section). The majority of CYPs selected for our toolbox are reported to have differential degrees of promiscuity and relaxed substrate specificity, which prevents anticipating product formation upon oxyfunctionalisation. Thus, a non-directed GC/MS-based metabolomics approach was used to investigate the chemical nature and regioselectivity of the CYP oxyfunctionalisation reactions (Methods Section). Our non-directed GC/MS screening detected a wide variety of oxyfunctionalised products. (Supplementary Tables S1–3 from which up to 70% could be conveniently identified (Supplementary Tables S4–5).

The activity of the CYP toolbox is summarized in Fig. 1. Conversion of the 15 different monoterpene substrates (**1**–**15**) studied is displayed in Fig. 1a and Supplementary Fig. S1–15. Figure 1b–d summarises the product landscape upon oxyfunctionalisation, including identified (**16**–**68**) and non-identified products (**u1**–**u27**). The entire list of product names can be found in the online Supplementary Tables S1–15. Our toolbox showed a remarkable range of activities against different monoterpene scaffolds with the best toolbox catalysts showing a wide range of product profiles and differential substrate selectivity: CYP101A1.1-3 (**4**, **6**–**8** and **9**–**15**), CYP102A1.Wt (**2**–**9** and **12**–**14**) and CYP153A6.Wt (**1**–**8**). This emphasises the capacity of these enzymes to form a comprehensive toolbox to widen monoterpenoid chemical diversity through oxyfunctionalisation. The substrate selectivity of the toolbox members can be rationalised by structural correlations between monoterpene scaffolds and the natural substrates of these CYPs: D-camphor (CYP101A1.Wt), fatty acids (CYP102A1.Wt) and *n*-alkanes (CYP153A6.Wt).

Initial studies focused on assessing the functional expression and activity of several bacterial CYPs, including: *Pseudomonas putida* P450_cam_ (CYP101A1), *Pseudomonas* sp. P450_terp_ (CYP108), *Bacillus megaterium* P450_BM3_ (CYP102A1), *Bacillus licheniformis* CYP102A7 and *Mycobacterium* HXN-1500. CYP153A6. CYP101A1 and CYP108 systems comprise the cytochrome P450 enzyme and two redox partners (redoxin and redoxin reductase). To investigate cofactor incorporation these components were expressed separately and purified. CYP108 was eliminated at this stage, as the redoxin 2S-2Fe cofactor was not incorporated. CYP101A1.Wt was reconstituted *in vitro* from purified proteins and was active with D-camphor as substrate. Functional expression of the CYP101A1 system in *E. coli* was achieved by constructing a polycistronic vector based on the JBEI-6411 backbone^2,8^ and previous studies^9,10^. Reconstituted CYP102A1.Wt and CYP102A7.Wt were active using substrate (*S*)-limonene **8** but CYP102A7 was subsequently excluded because of poor substrate conversion and functional stability^11^.

The CYP toolbox was diversified by addition of active site variants of CYP102A1.Wt and CYP101A1.Wt (see Table 1). The CYP102A1.1 triple variant (A264V/A328V/L437F) was selected from a small library reported previously^6^ as it can convert (*R*)-limonene (**7**) to (*R*)-perillyl alcohol **36**. CYP101A1 variants were designed based on structural information and previous engineering studies^5^. These variants combine different mutations (see Table 1) at four active site residues (Tyr96, Phe87, Leu244 and Val247). The Y96F mutation was included in all variants to better accommodate non-polar monoterpenes in the active site. Residues close to the heme cofactor (Leu244 and Val247), or on top of the binding pocket (Phe87), were altered with the aim of modifying reaction regioselectivity and substrate binding. Eight CYP101A1 variants were constructed. Among those incorporating the L244A mutation (CYP101A.4–8) only CYP101A1.6 showed enhanced conversion of monoterpenes compared to CYP101A1.Wt. The CYP101A1.1-3 variants showed high monoterpene conversion. The above variants showed small product regioselectivity differences, revealed by the ratio of (−)-*cis*-carveol (**46**) *vs* (−)-*trans*-isopiperitenol (**47**) or of (+)-*trans*-verbenol (**67**) *vs* (+)-myrtenol (**61**) after conversion of (*S*)-limonene (**8**) and (+)-α-pinene (**15**), respectively. Therefore, the diversity of oxyfunctionalised products achieved by the CYP toolbox relies mainly on the differential regioselectivity between CYP classes rather than through protein engineering within a specific class.

**Table 1:** CYP450 samples and variants employed in this study.

CYP450 family	Mutation	Name
CYP101A1	—	CYP101A1.Wt
	Y96F	CYP101A1.1
	Y96F, V247L	CYP101A1.2
	F87W, Y96F, V247L	CYP101A1.3
	Y96F, L244A	CYP101A1.4
	F87W, Y96F, L244A	CYP101A1.5
	Y96F, L244A, V247A	CYP101A1.6
	Y96F, L244A, V247F	CYP101A1.7
	Y96F, L244A, V247L	CYP101A1.8
CYP102A1	—	CYP102A1.Wt
	A264V, A328V, L437F	CYP102A1.1
CYP153A6	—	CYP153A6Wt

**Figure 2:**
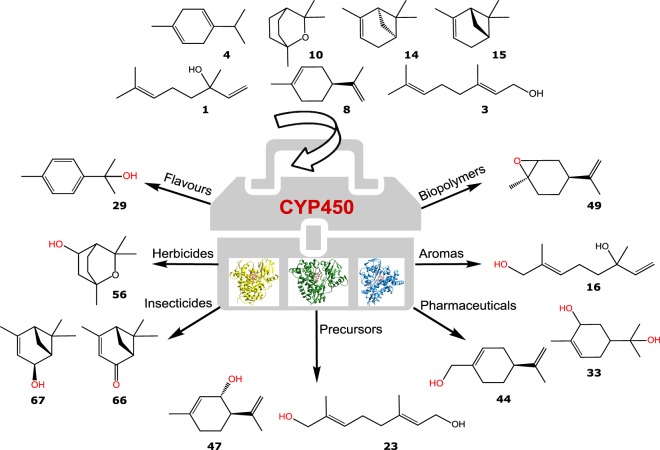
Key products formed by the CYP toolbox and their applications. Chemical structures from substrate scaffolds and products are shown. Miniatures of crystal structures from CYP101A1.Wt (*blue*) CYP102A1.Wt (*yellow*) and CYP153A7.Wt (similar sequence to CYP153A6.Wt, *green*) are shown (from PDB entries: 3wrj, 1bvy and 3rwl, respectively).

**Figure 3:**
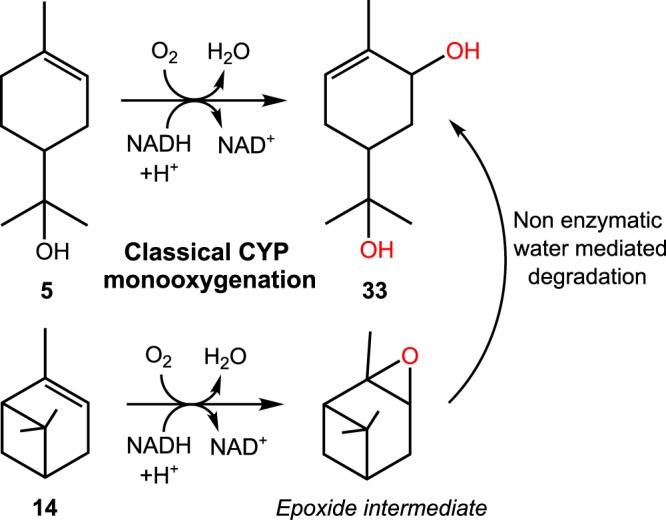
Putative routes of the CYP102A1.Wt-mediated conversion of α-terpineol **5** and (−)-α-pinene **14** to sobrerol **33**. The route from **5** to **33** is a classical CYP-like monooxygenation mechanism. The two step process from **14** includes a non-enzymatic water-mediated decomposition stage.

The observed products from monoterpene oxyfunctionalisation catalysed by the CYP toolbox include a variety of known pharmaceuticals, precursors, herbicides, flavours and aromas (Fig. 2). For example, we observed the production of sobrerol **33**, a known clinical expectorant and potential anti-cancer drug^12^. This product was obtained by biotransformations of CYP101A1 variants and CYP102A1.Wt with two structurally different scaffolds, namely (−)-α-pinene **14** and α-terpineol **5**, with the combination of CYP102A1.Wt and **14** being the best sobrerol **33** producing system. The reactions are thought to proceed via a conventional CYP-like monooxygenation mechanism, however the reaction with (−)-α-pinene likely includes the formation of an epoxide intermediate, followed by non-enzymatic water-mediated decomposition (Fig. 3)^13^.

Other pharmaceutical products obtained were (*4R*)- and (4*S*)-perillyl alcohol (**36** and **44**), which have known analgesic properties and a potential role in preventing Alzheimer’s disease^14,15^. The relaxed substrate enantioselectivity of CYP153A6.Wt was observed by the generation of the different enantiomeric products (**36** and **44**) during biotransformations with (*R*)- and (*S*)-limonene (**7** and **8**), respectively. The production *in vivo* of (4*R*)-perillyl alcohol **44** by metabolically engineering *E. coli* has been demonstrated previously *via* incorporation of a heterologous mevalonate pathway coupled with the *M. spicata* limonene synthase and CYP153A6^2,8^.

Biotransformations of CYP101A1.3 with **14** generated relatively small amounts of (−)-verbenone **63** and (−)-*trans*-verbenol **64**. These cytotoxic compounds are known to decrease the viability of either colon tumour cells or of normal cells, respectively, suggesting a potential role of **63** as a colon cancer drug^16^. Interestingly, reactions of CYP101A1.1 with the related isomer **15** generated high yields of (+)-verbenone **66** and (+)-*trans*-verbenol **67**, in line with previous studies^5,17^. However, it is not known whether **66** has a similar anti-tumour effect as its isomer **63**. These results are in line with studies using whole cells of *Picea abies*, where constitutive CYP enzyme(s) transformed racemic pinene into *cis*/*trans*- verbenol enantiomers (**64**/**65, 67**/**68**), with further transformations to **63** and **66**^18^. The latter compound is a well-known pheromone used to protect pine trees from attack by the mountain pine beetle^19^.

Biotransformations of CYP102A1.Wt with (*S*)-**8** generated *trans*-limonene-1,2-epoxide **49**, which is useful commercially as a monomer for copolymerisation with CO_2_ to form biodegradable polycarbonate^20^. In addition, both *cis*-carveol **46** and *cis*-isopiperitenol **48** were found in relatively small amounts. This is analogous to previous studies of CYP102A1.Wt with (*R*)-**7**, which showed a mixture of (4*R*)-limonene-1,2-epoxide, (4*R*)-limonene-8,9-epoxide and carveol^6,21^. Studies have shown that some terpene-derived polymers based on **7** and **8** have shown excellent properties for optoelectronic applications^22^. Therefore this CYP toolbox approach could provide valuable routes to existing and *de novo* biodegradable polymers.

The oxidation of (*S*)-**8** by CYP101A1.3 generated (−)-*trans*-isopiperitenol **47**, as reported before^17^, an intermediate in the *Mentha piperita* (peppermint) pathway to menthol isomers. Recent synthetic biology approaches towards the *in vitro* biosynthesis of (−)-menthol by recombinant *E. coli* extracts have been successful starting from pathway intermediates **47** and (*R*)-pulegone^23,24^. Therefore, the inclusion of a CYP generating **47** from (*S*)-**8** into existing recombinant pathways is a potentially important step forward towards complete microbial *in vivo* production of (−)-menthol from simple carbon sources^25^. 8-hydroxygeraniol **23** was also generated through the action of CYP153A6.Wt on geraniol **3**, in line with the terminal oxidation of aliphatic hydrocarbons observed previously^26,27^. This compound is part of the indole alkaloids loganin and secologanin biosynthesis routes in *Catharanthus roseus*^28^. These alkaloids are important due to their known antibacterial and anti-imflammatory properties^29^. Differently, 2,3- and 6,7-epoxy-geraniols were found after conversion of geraniol **3** by other CYP102A1 variants studied before^30^.

A variety of other monoterpenoid derivatives generated from the CYP toolbox are known to have diverse potential commercial applications. For example, the biotransformation of (+/−)-linalool **1** by CYP153A6.Wt generated 8-hydroxylinalool **16**, a known precursor of valuable fragrance additives such as lilac alcohols^29^. This activity was reported before for CYP111 as the first committed step for linalool **1** utilisation as sole carbon source in *Pseudomonas incognita*^31^. The oxidation of monoterpenoids γ-terpinene **4** generated *p*-cymen-8-ol **29**, a flavouring agent which contributes to the off-flavors in lemon juice^32^. Other products have known phytotoxic properties, such as 3-*exo*-hydroxy-1,8-cineole **56**, generated from the action of enzymes CYP101A1.1-3 on 1,8-cineole **10**. The hydroxylation of 1,8-cineole **10** by CYPs introduces several chiral centres which makes difficult the identification of reaction products^33–35^.

Our CYP toolbox provides an important repository of biocatalytic parts for synthetic biology, metabolic engineering and biocatalysis projects where the expansion of chemical diversity of monoterpene hydrocarbon scaffolds is required. Several studies have reported the evaluation of different CYP libraries for oxyfunctionalisation of monoterpene substrates such as (*R*)-limonene (**7**) or (+)-α-pinene (**15**) using traditional targeted GC^5^ or GC/MS analysis^6^ developed on a case-by-case basis. However, such tailored methods do not allow one to conduct a comprehensive and systematic study of different CYPs with diverse substrates thus limiting the access to a broad CYP catalysts landscape. Here we present an advanced approach for broad profiling of enzyme activities^36^ and enzyme function discovery^37^, combining the power of biocatalysis and non-directed metabolomics. Metabolomics or the systematic investigation of the comprehensive profile of all metabolites in a biological substrate is particularly well suited for impartially monitoring metabolic transformations, defined as consumption of substrates and generation of products. Non-directed metabolomics is of particular interest when products derived from such biotransformations cannot be anticipated, as in the case of the CYP library studied here. However, non-directed GC-MS metabolomics experiments produce large volumes of spectral data requiring essential data processing steps to uncover biological findings enclosed in such raw data. In this regard, an automated open-data and open-source GG-MS data analysis workflow has been implemented for rapid unbiased comparison of chromatograms. This consists of five automated sequential steps: *(i)* peak detection at the total ion count level for each chromatogram; *(ii)* matching and aligning homologous peaks across chromatograms; *(iii)* peak integration and normalization to corresponding internal standard; *(iv)* assessment of both substrate conversion and product landscape formation; *(v)* extraction of consensus spectra and computation of retention indices for each peak; and finally *(vi)* compound identification which requires manual curation and intensive interpretation of the observed mass spectra. In conclusion we report, for the first time, a comprehensively characterised CYP toolkit for metabolic pathway engineering and cascade biocatalysis to produce value added terpenoid products and terpene-based small molecule libraries for diverse applications.

## Methods

### Cell cultures, chemicals and commercial enzymes

Culture media for *E. coli* was obtained from Formedium. Cells were routinely grown in Lysogenic Broth (LB) or on LB agar plates including antibiotics, which were supplemented as appropriate (ampicillin, 100 μg·mL^−1^; kanamycin, 50 μg·mL^−1^). Cloning and plasmid propagation was performed using *E. coli* DH5α (New England Biolabs, NEB). The following chemicals and enzymes were purchased from Sigma: linalool, geraniol, (*E*/*Z*)-β-ocimene, (*R*)- and (*S*)-limonene, γ-terpinene, terpinolene, α-terpineol, (+)- and (−)-α-pinene, (+)- and (−)-β-pinene, (+)-fenchol, *sec*-butyl-benzene, ethyl-acetate, and glucose dehydrogenase (*Pseudomonas sp*.). (+)-car-3-ene and 1,8-cineole were purchased from TCI Chemicals. Information about the authentic standards used can be found in Supplementary Table S17.

### General molecular cloning

Cytochrome CYP153A6 and its redox partners encoded on the plasmid pJBEI-6411 (Addgene plasmid #47050) were a gift from Prof. Taek Soon Lee^2^. CYP450_cam_ (CYP101A1, variant C334A, referred to as Wt in this manuscript) and its redox partners PdR and PdX (variant C73S/C85S) were synthesised, codon optimised for *E. coli* and cloned into pETM11 or pET21b expression vectors (GeneArt, Life Technologies). Self-sufficient CYP450_BM3_ (CYP102A1) encoded in pET15b was kindly donated by Prof. Andrew Munro (The University of Manchester). A plasmid carrying CYP101A1.Wt together with its redox partners, PdR and PdX, was constructed and organized in an operon under the control of a single P_BAD_ promoter. Each gene was amplified via PCR from the corresponding expression vectors, while pJBEI-6411 was linearized by inverse PCR. The assembly of three genes/parts into one operon was performed using overlap-extension PCR (OE-PCR)^38^. Finally, pJBEI-6411 backbone and the fused OE-PCR products were assembled into one vector by In-Fusion^®^ cloning (Takara). The same procedure was employed to assemble CYP102A1.Wt into the pJBEI-6411 backbone. CYP101A1 and CYP102A1 variants were prepared using QuikChange^®^ site-directed mutagenesis (Stratagene). All constructs were confirmed by sequencing. All primers, PCR details, variants details, and pJBEI-6411-CYP101A1 map can be found in online Supplementary Methods.

### Protein expression and characterization

Single colonies were used to inoculate 50 mL of LB medium supplemented with the corresponding antibiotic. Those overnight cultures were used as starting material to inoculate large volume media (500 mL). All CYPs were grown on TB media supplemented with 15 μM FeCl_3_ at 37 °C until the optical density at 600 nm (OD_600_) reached 0.8–1 at which point 500 μM δ-aminolevulinic and 50 mM arabinose were added to support protein expression. After induction, cells were grown for 20–24 hours at 25 °C (CYP101A1 and CYP102A1) or 20 °C (CYP153A6) and collected by centrifugation at 4 °C. Cell pellets were resuspended (200 mg·mL^−1^) in 50 mM potassium phosphate buffer pH 7, 200 mM NaCl and 10% glycerol. Cells were lysed using a constant cell disruptor system (Constant Systems Ltd, UK). Protease inhibitor tablets (Sigma) were added to the cell lysates to avoid enzymatic degradation of over-expressed proteins. Cell lysates were immediately used for biotransformation or kept frozen at −80 °C. Heme iron thiolate coordination was established by formation of the Fe(II)CO complex at 450 nm after dithionite-reduction and bubbling with carbon monoxide gas. CYPs concentrations were calculated using the extinction coefficient ε_450_ = 96,000, as described previously^39^.

### Enzymatic biotransformations

Reactions were performed in 4 mL screw PTFE liner cap vials. The following 1 mL biotransformation mixtures (contained in 50 mM potassium phosphate buffer pH 7, 200 mM NaCl and 10% glycerol) were used: cell lysate (1.5 μM P450 final concentration), 0.2 mM NADH/NADPH, 5 mM monoterpene substrate, 30 mM glucose and 10 U·mL^−1^ glucose dehydrogenase. Enzymatic biotransformations were left shaking overnight at 25 °C.

### Oxyfunctionalisation activity-based metabolomic profiling

In order to detect and quantify unexpected products formed upon oxyfunctionalisation, an untargeted GC/MS metabolomics analysis was applied for each one of the individual fifteen substrates. Thus, for each substrate, we created an experimental batch containing vials from triplicate biotransformations of each CYP in our toolkit together with their respective blank (lysate free) and control (CYPs free) reactions. Following biotransformations, vials were cooled for 15 min at 4 °C and subsequently mixed with ethyl acetate (1:1) (containing 0.01% *sec*-butyl benzene used as internal standard for GC/MS analysis). Then, they were vigorously vortexed (1 min), centrifuged (14,000 rpm, 5 min, 4 °C) and dried over anhydrous MgSO_4_. 1 μL of the resulting mixture was automatically injected into a GC/MS system (Agilent Technologies 7890B GC and 5977 A MSD) equipped with DB-WAX column (30 m × 0.32 mm i.d., 0.25 μM film; Agilent Technologies). For each experimental batch an external standard containing C8-C20 alkanes was injected to further determine retention indexes (RI). GC/MS conditions were as follows: the injector temperature was set at 240 °C with a split ratio of 20:1 and the helium carrier flow-rate was kept constant at 2 mL·min^−1^; the oven temperature was 50 °C (1 min held), increased to 68 °C at 5 °C·min^−1^ rate (2 min held), and increased to 240 °C at 25 °C·min^−1^ (2 min held); the ion source temperature was set to 230 °C and it was operated in the electron impact ionization mode (70 eV); mass spectra were recorded at 6.6 scans·s^−1^ at mass scanning range from m/z 50–250 after a solvent delay of 2.20 min. For each batch an external standard containing C8-C20 alkanes was injected to further determine Kovats RI. Raw GC/MS files were converted to open-data format mzXML^40^ using Proteowizard^41^ and further data analysis was conducted using open-source R packages^42,43^. Peak peaking was performed at the total ion count (TIC) level in each chromatogram. Subsequently, homologous peaks were matched and aligned across samples within the same batch. Peaks were integrated and normalized to their corresponding internal standard peak area. To guarantee biological consistency, only those product peaks appearing in all sample triplicates and which median area was higher than the corresponding area in negative controls or blank reactions, were further considered. Product identification was conducted by comparison to reference spectra on the MS NIST library (level 2 annotation^44^) and/or using authentic standards (level 1 annotation^44^) as shown in online Supplementary Table S18. Substrate conversion (%) was calculated as per equation (1), where *S*_*x*_ and *S*_*c*_ represent the substrate peak area from each biotransformation and from the negative control, respectively.

1 $$Conv=100-(\frac{Sx}{Sc})\ast 100$$

## Electronic supplementary material

Supplementary Information

## Data Availability

All data generated or analysed during this study are included in this published article and its Supplementary Information.
