# Supplementary material for: A Toolbox for Diverse Oxyfunctionalisation of Monoterpenes
Source: Sci Rep. 2018 Sep 26;8:14396. doi: 10.1038/s41598-018-32816-1 (PMC6158249; doi:10.1038/s41598-018-32816-1)
Supplement: Supplementary file 1 — Supplementary Information [file 41598_2018_32816_MOESM1_ESM.pdf]

A Toolbox for Diverse Oxyfunctionalisation of Monoterpenes

Aitor Hernandez-Ortega, Maria Vinaixa, Ziga Zebec, Eriko Takano, Nigel S. Scrutton

Supplementary Information

Supplementary Results

Additional Information from the screening studies:
- **Supplementary Figures S1-15**: substrate conversion by sample
- **Supplementary Tables S1-15**: detailed product information

Supplementary Table S16: Mass Spectra of non-identified products.

Supplementary Table S17: Authentic standards used listed by retention time.

Supplementary Table S18: Product identification against NIST MS library and/or authentic standards.

Supplementary Methods
Supplementary Table S19: Basic information about primers.

Supplementary Table S20: Primers sequences.

Supplementary Figure S16: pJBEI-6411 construct map.

Supplementary Results

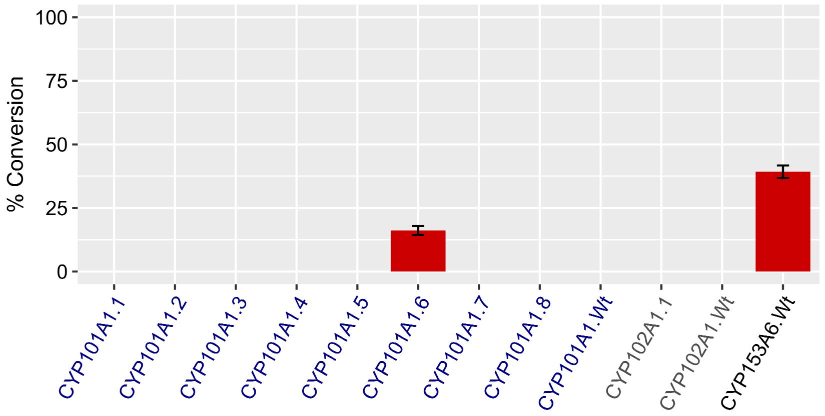

Supplementary Figure S1. Conversion % of (+/-/)-Linalool (1) by CYPs library.
Mean and standard deviation (error bars) shown.

Supplementary Table S1. Products formed after conversion of (+/-/)-Linalool (1) by CYPs library

Name (number)	SMILES	RT	RI	Normalised area (x100)	
				CYP101A1.6	CYP153A6.Wt
cis-Linalool oxide (**19**)	C[C@]1(CC[C@@H](O1)C(C)(C)O)C=C	9.04	1468	41.1±2.9	1.5±0.1
trans-Linalool oxide (**18**)	C[C@]1(CC[C@H](O1)C(C)(C)O)C=C	9.28	1621	24.3±2.4	1.8±0.1
Dihydrolinalool (**17**)	CCC(C)(CCC=C(C)C)O	9.76	1546	6.4±0.3	4.7±0.2
cis-Pyranoid linalool oxide (-)	CC1(C(CCC(O1)(C)C=C)O)C	10.96	1764	6.9±0.5	
trans-Pyranoid linalool oxide (-)	CC1(C(CCC(O1)(C)C=C)O)C	11.07	1859	5.3±0.4	
8-Hydroxylinalool (**16**)	CC(=CCCC(C)(C=C)O)CO	13.29	2000	10.4±3.5	231.6±9.6

RT: Retention time (minutes). RI: Retention index. Normalised area (x100): (mean± s.d.) calculated as described in the methods section.

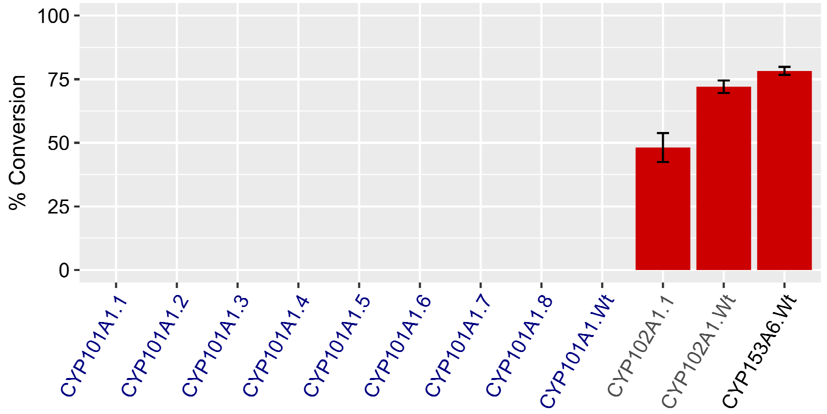

Supplementary Figure S2. Conversion % of (*E*/*Z*)-β-Ocimene (2) by CYPs library. Mean and standard deviation (error bars) shown.

Supplementary Table S2. Products formed after conversion of (*E*/*Z*)-β-Ocimene (2) by CYPs library

Name (number)	SMILES	RT	RI	Normalised area (x100)		
				CYP102A1.1	CYP102A1.Wt	CYP153A6.Wt
(Z)-Myroxide (**22**)	C/C(=C/CC1C(O1)(C)C)/C=C	9.24	1582	0.5±0.1	9.6±0.7	
(E)-Myroxide (**21**)	C/C(=C\CC1C(O1)(C)C)/C=C	9.40	1884	1.4±0.1	10.0±0.6	0.4±0.0
(3E,5E)-2,6-Dimethyl-3,5,7-octatrien-2-ol (**20**)	CC(=CC=CC(C)(C)O)C=C	11.32	1817	11.4±1.3	2.6±0.1	14.0±0.9
Oci (**u5**)		12.12	4973			64.7±2.6
Oci (**u4**)		12.26	2000	2.8±0.3		175.7±18.6
Oci (**u3**)		12.89	2000	2.2±0.1	17.3±2.4	
Oci (**u2**)		13.43	2000	23.4±1.2	12.4±0.6	13.4±0.4
Oci (**u1**)		13.81	2000	58.1±3.3	26.7±1.6	34.0±1.3

RT: Retention time (minutes). RI: Retention index. Normalised area (x100): (mean± s.d.) calculated as described in the methods section. Oci: unidentified (E/Z)-β -Ocimene products

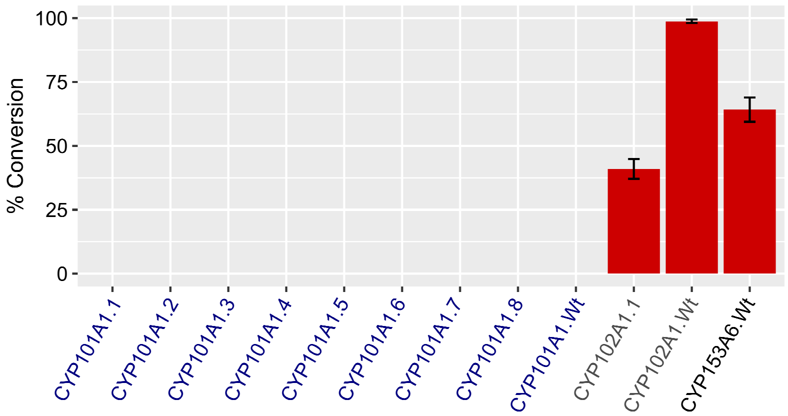

Supplementary Figure S3. Conversion % of Geraniol (3) by CYPs library.
Mean and standard deviation (error bars) shown.

Supplementary Table S3. Products formed after conversion of Geraniol (3) by CYPs library

Name	SMILES	RT	RI	Normalised area (x100)		
				CYP102A1.1	CYP102A1.Wt	CYP153A6.Wt
Ger (**u8**)		12.67	2000	7.9±0.8	125.3±2.0	13.0±2.7
Geranic acid (**24**)	CC(=CCCC(=CC(=O)O)C)C	13.41	2000	13.8±0.6	20.3±0.2	
Ger (**u7**)		13.59	2000	4.3±0.3	150.0±4.2	
Ger (**u6**)		14.42	2000	33.6±0.7	13.6±0.2	76.4±20.0
8-hydroxygeraniol (**23**)	CC(=CCO)CCC=C(C)CO	14.6	2000	102.9±4.5	13.8±0.3	229.6±15.0

RT: Retention time (minutes). RI: Retention index. Normalised area (x100): (mean± s.d.) calculated as described in the methods section. Ger: unidentified Geraniol products

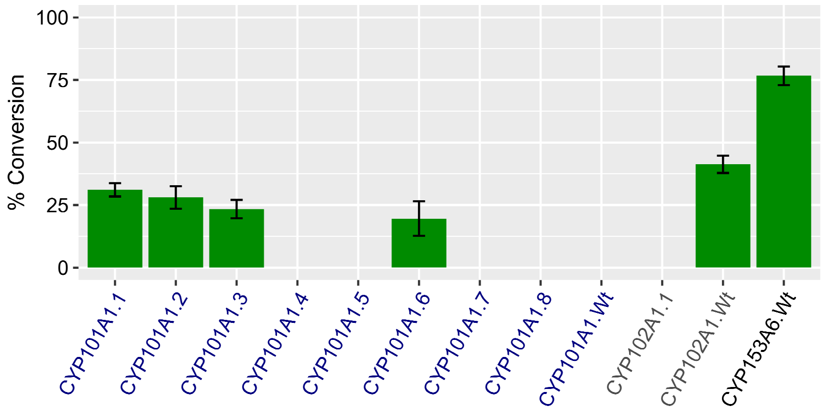

Supplementary Figure S4. Conversion % of γ-Terpinene (4) by CYPs library.
Mean and standard deviation (error bars) shown.

Supplementary Table S4. Products formed after conversion of γ-Terpinene (4) by CYPs library

Name (number)	SMILES	RT	RI	Normalised area (x100)*					
				**CYP101A1.1**	**CYP101A1.2**	**CYP101A1.3**	**CYP101A1.6**	**CYP102A1.Wt**	**CYP153A6.Wt**
p-cymene (-)	CC1=CC=C(C=C1)C(C)C	6.49	1368	1019.9±35.7	1155.8±50.2	1039.0±10.4	916.8±92.5	2055.8±315.4	13.1±2.4
p-cymenene (-)	CC1=CC=C(C=C1)C(=C)C	8.96	1445	96.4±2.2	74.0±6.7	41.2±1.7	21.1±2.2	152.5±2.2	
γ-Ter (**u9**)		9.6	1508	152.3±2.1	57.0±2.8	36.9±0.5	15.9±1.2	133.3±3.5	4.2±0.1
4-Isopropylbenzaldehyde (**31**)	CC(C)C1=CC=C(C=C1)C=O	11.19	2309					11.8±2.1	165.6±6.9
p-Mentha-1,4-dien-7-al (**30**)	CC(C)C1=CCC(=CC1)C=O	11.28	1806	20.5±1.0	42.6±1.0	46.3±1.7	14.0±1.8	15.7±1.3	998.4±41.9
p-Cymen-8-ol (**29**)	CC1=CC=C(C=C1)C(C)(C)O	11.47	1888	361.7±7.5	259.8±27.9	123.6±4.6	40.2±3.5	1101.6±74.7	
p-Mentha-1,4-dien-7-ol (**28**)	CC(C)C1=CCC(=CC1)CO	12.37	2000	11.2±0.4	9.6±3.4		83.1±6.0	36.4±3.7	2387.0±190.1
p-Cymen-7-ol (**27**)	CC(C)C1=CC=C(C=C1)CO	12.55	2000	20.7±2.3	61.9±88.0	10.8±1.2	139.9±13.6	197.1±6.6	147.6±31.3
Thymol (**26**)	CC1=CC(=C(C=C1)C(C)C)O	12.83	2000					89.2±3.3	
Carvacrol (**25**)	CC1=C(C=C(C=C1)C(C)C)O	12.95	2000	15.0±1.6	15.9±4.1	15.7±0.7		171.8±6.7	

RT: Retention time (minutes). RI: Retention index. Normalised area (x100): (mean± s.d.) calculated as described in the methods section. γ-Ter: unidentified γ-Terpinene products. *Normalised areas showing large numbers due to the partial overlapping of substrate and internal standard peaks which affects the peak relative area integration.

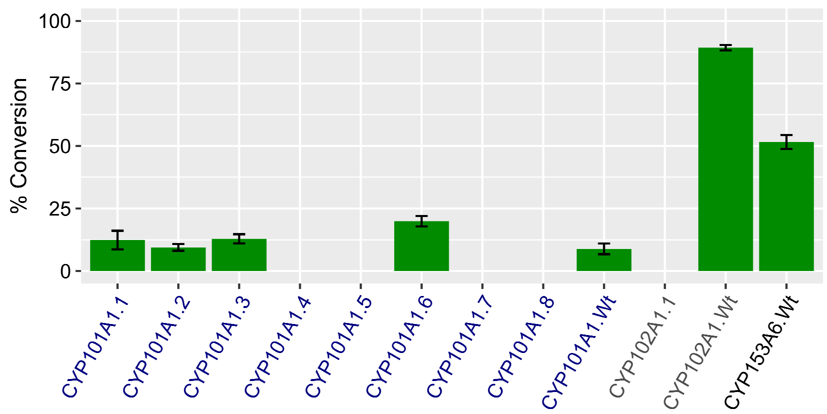

Supplementary Figure S5. Conversion of α-Terpineol (5) by CYPs library. Mean and standard deviation (error bars) shown.

Supplementary Table S5 Products formed after conversion of α-Terpineol (5) by CYPs library

**Product (name)**	**SMILES**	**RT**	**RI**	**Normalised area (x100)**						
				**CYP101A1.1**	**CYP101A1.2**	**CYP101A1.3**	**CYP101A1.6**	**CYP101A1.Wt**	**CYP102A1.Wt**	**CYP153A6.Wt**
α-Terp (**u11**)		12.1	2794	6.0±0.7	2.1±0.1	5.1±0.2	5.6±0.3	3.3±0.1	94.2±6.0	0.8±0.1
Sobrerol (**33**)	CC1=CCC(CC1O)C(C)(C)O	13.4	2000						19.2±1.0	
α-Terp (**u10**)		13.6	2000	5.6±0.2	5.0±0.4	6.0±0.3	5.5±0.4	5.5±1.2	37.2±2.0	7.9±0.7
7-hydroxyterpienol (**32**)	CC(C)(O)C1CCC(CO)=CC1	14.1	2000	3.6±0.4	4.0±0.1		5.2±0.1	4.5±0.3	32.5±0.2	157.9±6.5

RT: Retention time (minutes). RI: Retention index. Normalised area (x100): (mean± s.d.) calculated as described in the methods section. α-Terp: unidentified α-Terpineol products.

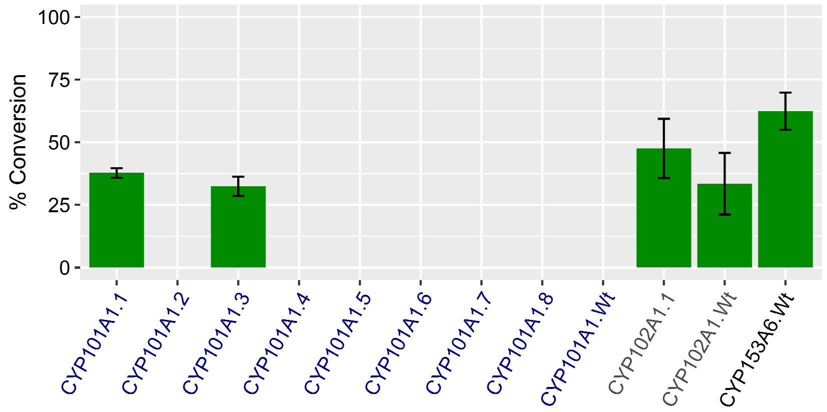

Supplementary Figure S6. Conversion of Terpinolene (6) by CYPs library.
Mean and standard deviation (error bars) shown.

Supplementary Table S6. Products formed after conversion of Terpinolene (6) by CYPs library

Name	SMILES	RT	RI	Normalised area (x100)					
				**CYP101A1.1**	**CYP101A1.2**	**CYP101A1.3**	**CYP101A1.6**	**CYP102A1.Wt**	**CY153A6.Wt**
p-Mentha-1,5,8-triene (-)	CC1=CCC(C=C1)C(=C)C	8.85	1424	4.3±0.1	6.2±0.5	6.4±1.1	3.0±1.4	1.7±0.3	1.4±0.7
Terp (**u14**)		12.28	2000	17.5±0.2	9.0±0.4	7.7±0.7	26.1±8.9	4.5±1.1	18.0±0.4
Terp (**u13**)		12.3	2000					3.5±0.7	39.5±0.8
p-Menthadien-7-ol (**34**)	CC(C)C1=CC=C(CC1)CO	12.53	2000						86.1±2.1
Terp (**u12**)		13.47	2000				7.1±0.6	26.5±5.0	

RT: Retention time (minutes). RI: Retention index. Normalised area (x100): (mean± s.d.) calculated as described in the methods section. Ter: unidentified Terpinolene products.

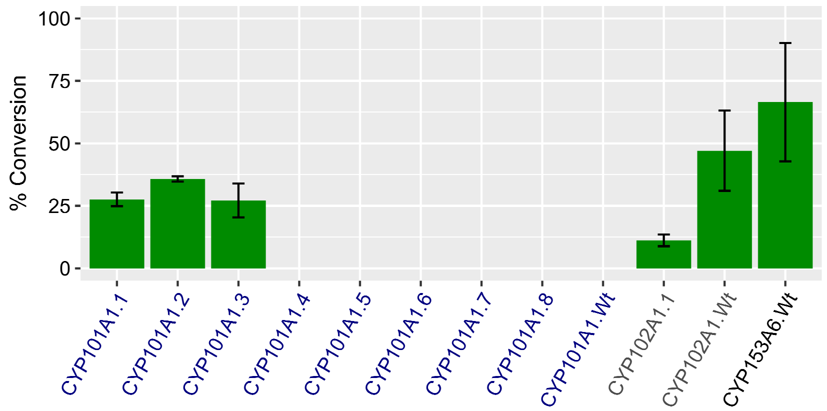

Supplementary Figure S7. Conversion of (*R*)-Limonene (7) by CYPs library.
Mean and standard deviation (error bars) shown.

Supplementary Table S7 .Products formed after conversion of (*R*)-Limonene (7) by CYPs library

Name (Number)	SMILES	RT	RI	Normalised area (x100)					
				CYP101A1.1	CYP101A1.2	CYP101A1.3	CYP102A1.1	CYP102A1.Wt	CYP153A6.Wt
(+)-*cis*-Limonene-1,2-epoxide (**42**)	C=C([C@@H]1CC[C@@]2([H])OC2C1)C	9.04	1467	28.5±0.3	30.5±0.8	10.4±0.3	7.4±0.4	25.9±1.9	2.2±0.8
(+)-*trans*-Limonene-1,2-epoxide (**41**)	C=C([C@@H]1CC[C@]2([H])OC2C1)C	9.16	1514	3.5±0.0	3.7±0.1	3.4±0.1	5.8±0.1	10.8±0.7	2.6±0.5
D-Dihydrocarvone (-)	CC1CCC(CC1=O)C(=C)C	10.24	1603					2.0±0.1	0.5±0.3
trans-d-hydrocarvone (-)	CC1CCC(CC1=O)C(=C)C	10.25	1606	1.8±0.0	2.0±0.1		8.3±0.3		0.7±0.2
(+)-*cis*-Isopiperitenol (**40**)	CC1=C[C@H](O)[C@H](C(C)=C)CC1	11.03	1810					129.4±5.0	
(+)-*trans*-Isopiperitenol (**39**)	CC1=C[C@@H](O)[C@H](C(C)=C)CC1	11.05	1830	48.5±0.6	75.1±3.8	87.4±10.6	4.1±0.2		2.8±0.2
Perillyl aldehyde (-)	CC([C@@H]1CCC(C=O)=CC1)=C	11.22	2751				3.2±0.9		12.4±0.5
(+)-*cis*-Carveol (**38**)	CC([C@H]1CC=C([C@H](C1)O)C)=C	11.42	1854	20.1±0.4	21.0±0.9	8.5±0.6			6.5±0.3
(+)-*trans*-Carveol (**37**)	CC([C@H]1CC=C([C@@H](C1)O)C)=C	11.57	2003				11.4±0.2	26.5±0.2	
(+)-Perillyl alcohol (**36**)	CC([C@H]1CC=C([C@@H](C1)O)C)=C	12.18	2000	2.3±0.1	2.8±0.1	1.4±0.0	87.9±8.0	6.5±0.1	228.2±31.6
Limonene-1,2-diol (**35**)	CC([C@H]1CCC(C)(O)C(O)C1)=C	13.18	2000	10.5±0.3	11.4±0.5	4.9±0.2	14.8±5.2	29.6±0.3	4.0±0.2

RT: Retention time (minutes). RI: Retention index. Normalised area (x100) (mean± s.d.) calculated as described in the methods section.

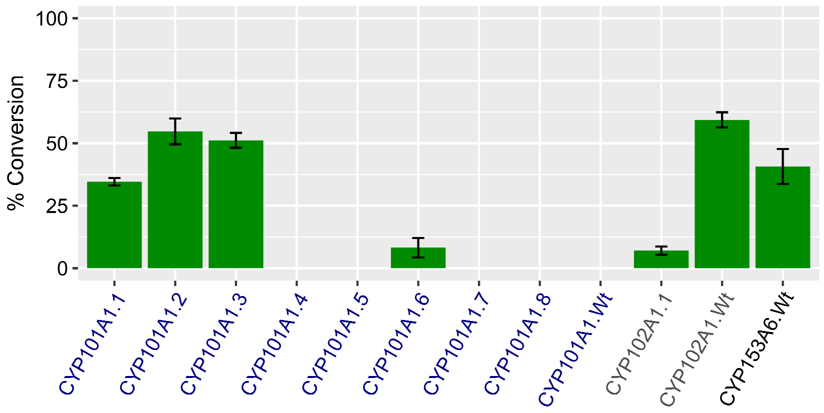

Supplementary Figure S8. Conversion of (*S*)-Limonene (8) by CYPs library.
Mean and standard deviation (error bars) shown.

Supplementary Table S8. Products formed after conversion of (*S*)-Limonene (8) by CYPs library

Name (number)	SMILES	RT	RI	Normalised area (x100)						
				CYP101A1.1	CYP101A1.2	CYP101A1.3	CYP101A1.6	CYP102A1.1	CYP102A1.Wt	CYP153A6.Wt
(-)-*cis*-Limonene-1,2- epoxide (**50**)	C=C([C@H]1CC[C@]2([H])OC2C1)C	9.04	1467	4.7±0.6	1.7±0.8	2.6±1.2	1.8±0.0	2.1±0.1	9.4±0.2	0.9±0.0
(-)-*trans*-Limonene-1,2-epoxide (**49**)	C=C([C@H]1CC[C@@]2([H])OC2C1)C	9.16	1514	3.4±0.1	3.3±0.3	2.6±0.1	0.6±0.0	1.3±0.0	68.2±0.9	0.4±0.0
(-)-*cis*-Isopiperitenol (**48**)	CC1=C[C@@H](O)[C@@H](C(C)=C)CC1	11.03	1810						46.6±1.0	
(-)-*trans*-Isopiperitenol (**47**)	CC1=C[C@H](O)[C@@H](C(C)=C)CC1	11.05	1830	112.0±3.6	156.9±12.7	187.3±3.3	21.6±0.6	2.3±0.2		1.5±0.2
Perillyl aldehyde (-)	CC([C@H]1CCC(C=O)=CC1)=C	11.22	2751					4.7±0.3		9.5±2.2
(-)-*cis*-Carveol (**46**)	CC([C@@H]1CC=C([C@@H](C1)O)C)=C	11.42	1854	35.0±1.3	53.4±4.2	17.5±0.8	3.9±0.1	6.9±0.1	41.3±1.0	3.2±0.1
α-Limonene diepoxide (**45**)	CC12CCC(CC1O2)C3(CO3)C	11.48	1891	6.8±0.4	9.7±0.5				63.2±2.9	
trans-Shisool (-)	CC(=C)C1CCC(CC1)CO	11.88	1966	1.5±0.2			1.2±0.1	4.0±0.2		2.5±0.3
(-)-Perillyl alcohol (**44**)	CC([C@H]1CCC(CO)=CC1)=C	12.18	2000	2.3±0.1	2.3±0.1	1.0±0.1	8.0±0.2	58.4±6.5	7.6±0.2	214.3±20.7
Limonene-1,2-diol (**43**)	CC([C@@H]1CCC(C)(O)C(O)C1)=C	13.18	2000	4.7±0.4	4.1±0.3	4.0±0.4	3.5±0.1	8.5±0.5	15.4±0.5	5.8±4.5

RT: Retention time (minutes). RI: Retention index. Normalised area (x100): (mean± s.d.) calculated as described in the methods section.

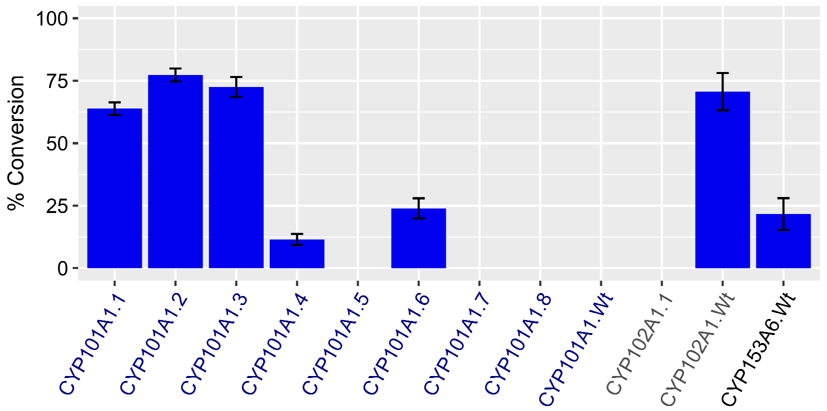

Supplementary Figure S9. Conversion of (+)-Carene (9) by CYPs library.
Mean and standard deviation (error bars) shown.

Supplementary Table S9. Products formed after conversion of (+)-Carene (9) by CYPs library

Name (Number)	SMILES	RT	RI	Normalised area (x100)						
				CYP101A1.1	CYP101A1.2	CYP101A1.3	CYP101A1.4	CYP101A1.6	CYP102A1.Wt	CYP153A6.Wt
p-Cymene	CC1=CC=C(C=C1)C(C)C	6.48	1368	14.4±0.8	14.7±1.6	15.5±0.3	7.7±0.2	11.9±0.3	1.9±0.4	
Car (**u17**)		9.06	1474	93.3±0.3	83.8±2.3	36.1±0.8	6.7±0.1	9.5±0.2	62.2±4.6	10.2±0.4
p-Mentha-1,5-dien-8-ol (**55**)	CC1=CCC(C=C1)C(C)(C)O	10.85	1719	165.8±1.5	184.8±4.5	242.0±1.9	32.3±0.8	149.6±4.2	46.6±5.1	14.5±0.6
p-Mentha-1,3-dien-8-ol (**54**)	CC1=CC=C(C(C)(O)C)CC1									
p-Mentha-1,5-dien-8-ol (**55**)	CC1=CCC(C=C1)C(C)(C)O	10.89	1734	105.4±3.8	134.0±6.7	92.1±1.7	10.3±0.2	23.3±0.9	14.7±2.7	
p-Mentha-1,3-dien-8-ol (**54**)	CC1=CC=C(C(C)(O)C)CC1									
Car (**u16**)		11.05	1838	48.6±3.6	69.1±5.5	50.3±1.1	4.5±0.3	8.7±0.4	7.4±0.8	3.3±0.3
m-Cymen-8-ol (**53**)	CC1=CC(=CC=C1)C(C)(C)O	11.44	1869	10.3±0.7	12.8±1.1	29.8±0.4	1.2±0.1	4.8±0.6	8.9±0.9	
Car-3-en-5-one (**52**)	CC1=CC(=O)C2C(C1)C2(C)C	11.87	1961	9.2±0.5	8.8±0.7	8.8±0.1	3.4±0.0	10.8±0.5	5.1±0.3	
Car (**u15**)		11.91	1989							174.6±3.8
3-Caren-10-ol (**51**)	OCC(CC12)=CCC2C1(C)C	11.95	2026	7.5±1.6	13.0±1.0	9.2±0.7	1.8±0.1	5.3±0.7	4.0±0.9	50.7±0.6
p-Cymen-7-ol (-)	CC(C)C1=CC=C(C=C1)CO	12.5	2000						1.2±0.1	6.3±0.6

RT: Retention time (minutes). RI: Retention index. Normalised area (x100): relative peak area (mean± s.d.) calculated as described in the methods section. Car: unidentified (+)-Carene products.

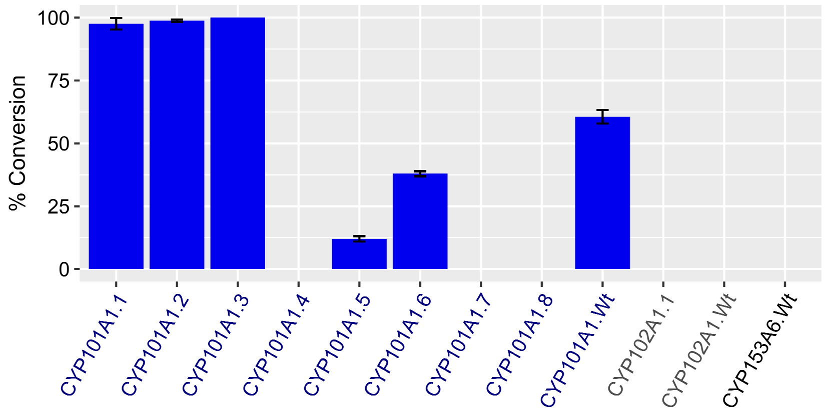

Supplementary Figure S10. Conversion of 1,8-Cineole (10) by CYPs library. Mean and standard deviation (error bars) shown.

Supplementary Table S10. Products formed after conversion of 1,8-Cineole (10) by CYPs library

Name (number)	SMILES	RT	RI	Normalised area (x100)					
				CYP101A1.1	CYP101A1.2	CYP101A1.3	CYP101A1.5	CYP101A1.6	CYP101A1.Wt
Cin (**u19**)		10.5	1693	25.1±13.6	16.0±1.0	53.4±2.4	1.3±0.1	2.9±0.1	6.9±0.6
2-Hydroxycineole (**57**)	CC1(C2CCC(O1)(C(C2)O)C)C	11.5	1983	50.6±5.9	33.9±1.8	7.9±0.4	2.6±0.1	20.1±0.4	67.8±3.5
3-*exo*-Hydroxy-1,8-cineole (**56**)	CC1(C)[C@@H]2CC[C@@](C[C@H]2O)(C)O1	11.8	1920	275.6±32.4	300.3±11.5	288.2±9.6	63.5±3.4	142.3±0.5	171.1±8.6
Cin (**u18**)		11.9	1973	62.7±8.3	81.3±3.1	69.6±3.4	7.1±0.4	22.4±1.2	48.7±1.8

RT: Retention time (minutes). RI: Retention index. Normalised area (x100): (mean± s.d.) calculated as described in the methods section. Cin: unidentified 1,8-Cineole products.

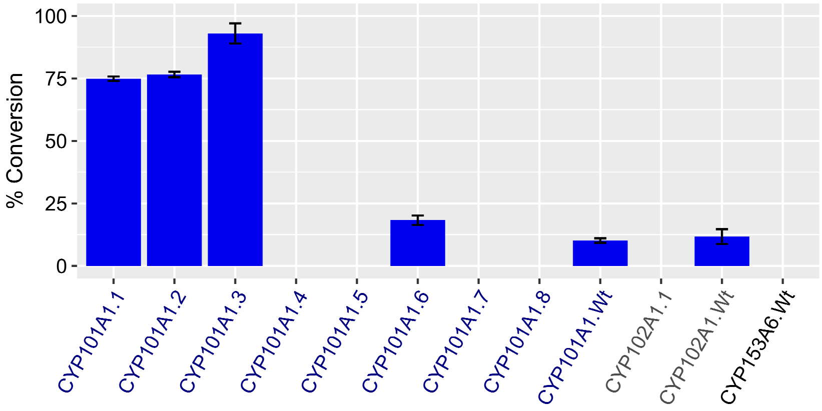

Supplementary Figure S11. Conversion of (+)-Fenchol (11) by CYPs library.
Mean and standard deviation (error bars) shown.

Supplementary Table S11 Products formed after conversion of (+)-Fenchol (11) by CYPs library

Name (Number)	SMILES	RT	RI	Normalised area (x100)					
				CYP101A1.1	CYP101A1.2	CYP101A1.3	CYP101A1.6	CYP101A1.Wt	CYP102A1.Wt
Fenchone (-)	CC1(C2CCC(C2)(C1=O)C)C	8.54	2094	3.9±0.1	2.5±0.1	1.3±0.4	3.9±0.1	1.6±0.1	7.8±0.3
Fen (**u21**)		12.78	2000	53.4±12.1	54.3±21.9	59.0±6.4	9.9±1.4	11.0±5.3	1.6±0.1
Fen (**u20**)		13.28	2000	378.8±21.3	443.3±108.4	409.2±30.0	108.0±5.7	81.0±15.6	46.2±2.4

RT: Retention time (minutes). RI: Retention index. Amount: relative peak area (mean± s.d.) calculated as described in the methods section. Fen: unidentified (+)-Fenchol products.

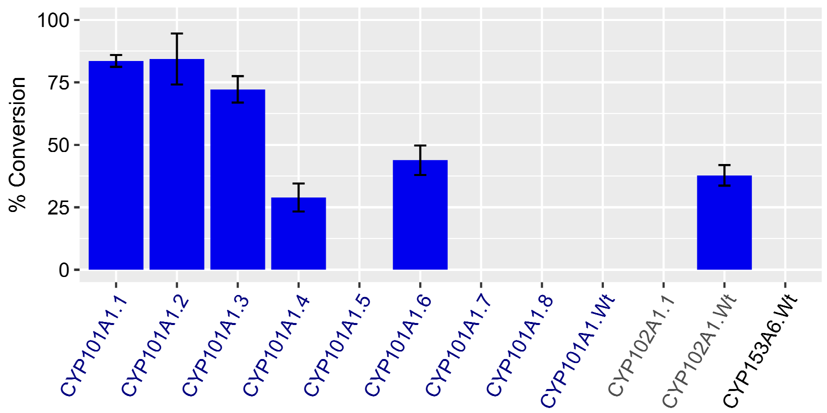

Supplementary Figure S12. Conversion of (-)-β-Pinene (12) by CYPs library.
Mean and standard deviation (error bars) shown.

Supplementary Table S12. Products formed after conversion of (-)-β-Pinene (12) by CYPs library

Name (number)	SMILES	RT	RI	Normalised area (x100)					
				CYP101A1.1	CYP101A1.2	CYP101A1.3	CYP101A1.4	CYP101A1.6	CYP102A1.Wt
(-)-*trans*-Pinocarveol (**60**)	CC1(C)[C@@H]2C[C@H]1C([C@@H](O)C2)=C	10.5	1634				12.3±1.0	13.6±0.4	56.6±4.5
(-)-Myrtenol (**59**)	CC1([C@@H]2C[C@H]1C(CO)=CC2)C	11.2	4462	9.4±2.0	7.4±1.4	1.7±0.2		6.2±0.4	
(-)-*cis*-Myrtanol (-)	CC1(C)[C@H]2CC[C@@H](CO)[C@@H]1C2	11.6	1934	4.9±0.9			2.7±0.4	3.0±0.2	4.3±0.3
Perillyl alcohol (**58**)	CC(=C)C1CCC(=CC1)CO	12.2	1905	22.0±1.5	17.2±2.4	8.3±1.7	9.5±0.1	9.7±0.4	3.2±0.5
p-menthadien-7-ol (**34**)	CC©C1=CC=C(CC1)CO	12.5	1976	36.8±2.0	28.4±4.4	21.8±3.4	14.6±0.1	14.5±0.6	5.4±0.7
7-Hydroxy-terpineol (**32**)	CC(C1CCC(CO)=CC1)(O)C	14.1	2000	212.4±19.3	160.9±25.4	126.3±15.5	97.4±2.6	88.6±3.9	148.5±14.5

RT: Retention time (minutes). RI: Retention index. Normalised area (x100): (mean± s.d.) calculated as described in the methods section.

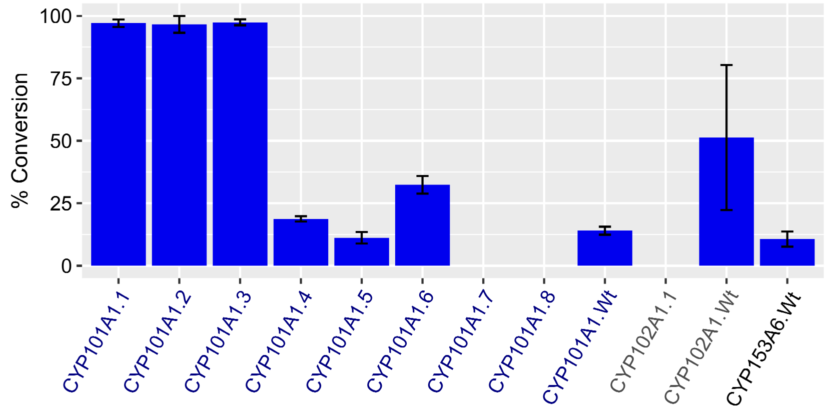

Supplementary Figure S13. Conversion of (+)-β-Pinene (13) by CYPs library.
Mean and standard deviation (error bars) shown.

Supplementary Table S13. Products formed after conversion of (+)-β-Pinene (13) by CYPs library

Name (number)	SMILES	RT	RI	Normalised area (x100)								
				**CYP101A1.1**	**CYP101A1.2**	**CYP101A1.3**	**CYP101A1.4**	**CYP101A1.5**	**CYP101A1.6**	**CYP101A1.Wt**	**CYP102A1.Wt**	**CYP153A6.Wt**
(+)-*trans*-Pinocarveol (**62**)	CC1(C)[C@H]2C[C@@H]1C([C@H](O)C2)=C	10.5	1739	6.5±1.5	2.8±0.7		18.4±0.4	8.4±0.1	22.4±0.3	1.5±0.1	4.9±2.2	42.2±0.5
(+)-Myrtenol (**61**)	CC1([C@H]2C[C@@H]1C(CO)=CC2)C	11.2	4869	10.8±0.7	6.6±0.6	1.0±0.3	4.4±0.1	2.9±0.1	4.8±0.1	2.2±0.1	2.0±0.6	5.2±0.2
(+)-*cis*-Myrtanol (-)	CC1(C)[C@@H]2CC[C@H](CO)[C@H]1C2	11.6	2046	4.6±0.7	2.3±0.2		2.4±0.1	2.0±0.1	1.8±0.1	2.8±0.2	2.1±0.7	1.0±0.1
Perillyl alcohol (**58**)	CC(=C)C1CCC(CO)=CC1	12.2	2000	21.3±0.3	22.1±0.5	12.3±0.2	3.4±0.1	2.4±0.1	4.3±0.1	3.1±0.1		
p-Menthadien-7-ol (**34**)	CC(C)C1=CC=C(CC1)CO	12.5	2000	38.1±0.7	39.4±1.0	26.7±0.6	5.6±0.0	3.8±0.2	7.4±0.1	5.1±0.0	2.6±0.7	
7-Hydroxy-terpineol (**32**)	CC(C1CCC(CO)=CC1)(O)C	14.1	2000	215.1±0.1	229.2±4.7	152.7±3.9	34.9±1.8	25.4±0.8	45.7±1.3	30.2±1.0	57.0±30.3	6.2±0.5

RT: Retention time (minutes). RI: Retention index. Normalised area (x100): (mean± s.d.) calculated as described in the methods section.

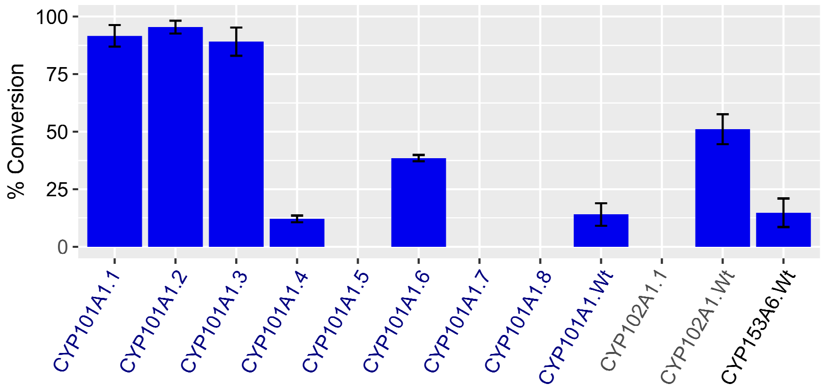

Supplementary Figure S14. Conversion of (-)-α-Pinene (14) by CYPs.
Mean and standard deviation (error bars) shown.

Supplementary Table S14. Products formed after conversion of (-)-α-Pinene (14) by CYPs library

Name (number)	SMILES	RT	RI	Normalised area (x100)							
				CYP101A1.1	CYP101A1.2	CYP101A1.3	CYP101A1.4	CYP101A1.6	CYP101A1.Wt	CYP102A1.Wt	CYP153A6.Wt
(-)-*cis*-verbenol (**65**)	CC1=C[C@H](O)[C@H]2C[C@@H]1C2(C)C	10.53	1735	59.3±11.9	42.6±4.5	27.9±4.5	43.9±1.7	27.3±1.0	7.4±0.3	8.2±0.3	2.9±0.0
(-)-*tras*-verbenol (**64**)	CC1=C[C@@H](O)[C@H]2C[C@@H]1C2(C)C	10.66	2007	52.4±7.3	44.6±2.7	38.4±4.0	53.8±1.6	52.2±3.1	13.3±0.4	47.9±1.2	15.4±0.3
(-)-Verbenone (**63**)	CC1=CC([C@H]2C[C@@H]1C2(C)C)=O	10.83	1713	39.8±0.7	55.5±1.3	100.5±5.2	22.3±1.1	36.8±2.3	2.3±0.2	5.2±0.2	2.5±0.0
(-)-Myrtenol (**59**)	CC1([C@@H]2C[C@H]1C(CO)=CC2)C	11.24	4661	17.8±2.0	10.1±0.9	15.6±0.3	14.6±0.4	9.8±0.2	8.6±0.4	5.6±0.1	21.0±0.3
(-)-α-Pin (**u25**)		11.69	3889	33.4±0.4	32.7±0.6	12.4±0.6	1.8±0.1	3.2±0.8	1.9±0.6	9.0±0.4	
Camphostene (-)	CC1(C2CC1C(C(=O)C2)(C)O)C	12	2111	17.9±0.5	8.7±0.2	4.6±0.2		4.2±2.2			
(-)-α-Pin (**u24**)		12.15	2000	15.9±1.1	11.6±0.6	43.4±1.1	7.5±0.3	12.1±1.6	26.5±0.9	4.3±0.2	
Sobrerol (**33**)		13.35	2000	45.6±6.4	50.1±4.2	33.1±4.6	14.6±0.4	26.3±7.8	17.5±2.7	186.8±14.5	46.0±0.5
(-)-α-Pin (**u23**)		14.21	2000	26.8±18.0	15.6±1.7	25.1±9.7		34.9±28.0	20.7±12.4		
(-)-α-Pin (**u22**)		15	2000	34.1±9.3	28.6±1.0	37.0±10.4	16.6±1.0	49.8±20.9	16.2±6.8		

RT: Retention time (minutes). RI: Retention index. Normalised area (x100): (mean± s.d.) calculated as described in the methods section. (-)-α-Pin: unidentified (-)-α-Pinene products.

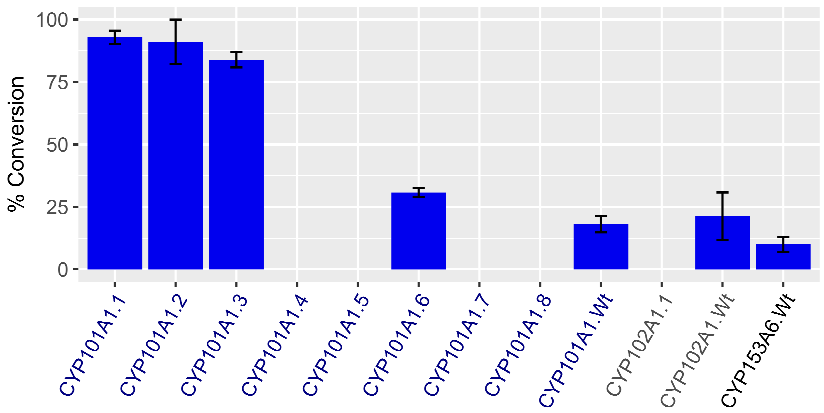

Supplementary Figure S15. Conversion of (+)-α-Pinene (15) by CYPs library.
Mean and standard deviation (error bars) shown.

Supplementary Table S15. Products formed after conversion of (+)-α-Pinene (15) by CYPs library

Name (Number)	SMILES	RT	RI	Normalised area (x100)						
				**CYP101A1.1**	**CYP101A1.2**	**CYP101A1.3**	**CYP101A1.6**	**CYP101A1. Wt**	**CYP102A1. Wt**	**CYP153A6. Wt**
(+)-Myrtenal (-)	CC1([C@H]2C[C@@H]1C(C=O)=CC2)C	10.4	1640	1.9±0.4	1.9±0.3	0.5±0.0		0.6±0.0		
(+)-*cis*-Verbenol (**68**)	CC1=C[C@@H](O)[C@@H]2C[C@H]1C2(C)C	10.6	1758	34.0±0.3	46.8±7.7	21.1±1.6	24.0±0.7	12.2±0.7	7.5±2.0	1.5±0.1
(+)-*tras*-verbenol (**67**)	CC1=C[C@H](O)[C@@H]2C[C@H]1C2(C)C	10.7	2091	194.3±6.5	130.4±24.9	77.5±6.5	47.2±1.9	12.7±1.2	15.6±2.7	6.5±0.3
(+)-Verbenone (**66**)	CC1=CC([C@@H]2C[C@H]1C2(C)C)=O	10.8	1715	32.5±1.4	90.6±5.2	145.9±3.1	23.3±1.6	2.4±0.3	1.8±0.0	1.1±0.2
(+)-Myrtenol (**61**)	CC1([C@H]2C[C@@H]1C(CO)=CC2)C	11.3	1802	68.4±6.3	49.0±8.2	9.0±0.6	7.3±0.2	10.7±0.3	6.5±1.1	9.5±0.1
Sobrerol (**33**)	CC1=CCC(CC1O)C(C)(C)O	13.4	2000	30.9±13.6	17.4±3.3	8.7±0.5	9.6±1.0	7.6±1.3	15.5±4.0	20.4±1.4
(+)-α-Pin (**u27**)		13.5	2000	27.6±26.4	12.7±1.9	9.3±1.3		6.7±1.4		
(+)-α-Pin (**u26**)		15	2000	31.0±23.9	27.1±2.8	40.7±0.8	13.2±1.0	8.7±2.0		

RT: Retention time (minutes). RI: Retention index. Normalised area (x100): (mean± s.d.) calculated as described in the methods section. (+)-α-Pin: unidentified (+)-a-Pinene products.

Supplementary Table S16. Mass Spectra of non-identified products			
**Substrate used**	**Product Number**	**Product Retention Time (min)**	**EI-MS, m/z (relative intensity)**
(*E*/*Z*)-β-Ocimene (**2**)	**u1**	12.12	121 (21), 119 (30), 117 (5), 106 (8), 105 (25), 103 (6), 97 (6), 95 (14), 94 (79), 93 (98), 92 (60), 91 (68), 84 (21), 83 (6), 81 (18), 80 (23), 79 (100), 78 (10), 77 (49), 71 (6), 69 (8), 68 (7), 67 (28), 66 (6), 65 (13), 55 (25), 53 (22), 51 (9).
	**u2**	12.26	134 (8), 121 (30), 119 (32), 117 (5), 109 (6), 106 (7), 105 (23), 103 (6), 97 (14), 96 (7), 95 (12), 94 (41), 93 (84), 92 (28), 91 (61), 84 (11), 83 (6), 82 (6), 81 (25), 80 (53), 79 (100), 78 (9), 77 (47), 71 (7), 69 (8), 68 (9), 67 (29), 66 (6), 65 (13), 57 (5), 55 (27), 53 (21), 51 (8).
	**u3**	12.89	153 (7), 128 (6), 127 (10), 115 (6), 111 (6), 110 (47), 109 (14), 99 (8), 98 (11), 97 (23), 95 (9), 85 (14), 84 (100), 83 (58), 82 (26), 81 (55), 79 (11), 73 (6), 72 (25), 71 (94), 70 (28), 69 (37), 68 (5), 67 (11), 59 (86), 57 (15), 56 (14), 55 (96), 54 (5), 53 (11).
	**u4**	13.43	137 (8), 119 (7), 109 (23), 107 (7), 101 (19), 97 (8), 95 (11), 94 (11), 93 (15), 91 (15), 84 (6), 83 (36), 82 (12), 81 (100), 80 (10), 79 (72), 77 (17), 73 (7), 72 (70), 71 (19), 70 (34), 69 (24), 68 (6), 67 (16), 65 (7), 59 (75), 57 (29), 55 (31), 53 (16), 51 (5).
	**u5**	13.81	101 (24), 84 (8), 83 (61), 73 (6), 71 (13), 70 (100), 69 (22), 67 (5), 59 (8), 57 (6), 56 (6), 55 (98), 53 (11).
Geraniol (**3**)	**u6**	12.67	111 (5), 110 (6), 109 (25), 98 (5), 97 (29), 95 (10), 94 (11), 93 (8), 91 (5), 87 (11), 86 (9), 85 (90), 84 (45), 83 (28), 82 (22), 81 (100), 80 (7), 79 (44), 77 (9), 72 (25), 71 (66), 70 (12), 69 (32), 68 (13), 67 (33), 65 (6), 59 (97), 57 (37), 56 (10), 55 (27), 53 (17).
	**u7**	13.59	86 (6), 85 (100), 83 (41), 69 (6), 68 (46), 67 (25), 57 (6), 55 (9), 53 (5).
	**u8**	14.42	137 (11), 135 (16), 121 (13), 119 (5), 111 (7), 109 (9), 108 (6), 107 (10), 97 (8), 95 (12), 94 (23), 93 (14), 91 (10), 85 (19), 84 (100), 83 (58), 82 (12), 81 (16), 80 (11), 79 (14), 77 (8), 71 (10), 69 (12), 68 (18), 67 (25), 65 (7), 57 (19), 56 (16), 55 (26), 53 (17), 51 (5).
γ-Terpinene (**4**)	**u9**	9.60	152 (26), 137 (46), 134 (11), 123 (15), 121 (5), 119 (40), 110 (12), 109 (100), 108 (6), 107 (12), 105 (7), 95 (27), 94 (10), 93 (28), 92 (6), 91 (34), 82 (9), 81 (50), 80 (5), 79 (35), 78 (5), 77 (27), 71 (5), 69 (10), 68 (7), 67 (69), 66 (5), 65 (12), 55 (23), 53 (14), 51 (7).
γ-Terpineol (**5**)	**u10**	12.09	137 (18), 126 (9), 112 (21), 111 (17), 109 (32), 108 (31), 97 (23), 95 (13), 94 (11), 93 (25), 91 (5), 84 (11), 83 (21), 82 (5), 81 (11), 79 (16), 77 (5), 72 (6), 71 (100), 70 (9), 69 (20), 68 (11), 67 (14), 59 (68), 58 (13), 57 (5), 55 (16), 53 (6).
	**u11**	13.57	152 (15), 137 (41), 119 (11), 110 (11), 109 (97), 108 (8), 97 (7), 96 (5), 95 (30), 94 (81), 93 (39), 91 (18), 84 (9), 83 (9), 82 (5), 81 (15), 80 (9), 79 (100), 77 (17), 71 (6), 70 (7), 69 (20), 67 (10), 65 (5), 59 (81), 55 (17), 53 (8).
Terpinolene (**6**)	**u12**	12.28	152 (31), 134 (28), 123 (6), 122 (10), 121 (100), 119 (36), 117 (6), 115 (5), 109 (8), 106 (8), 105 (29), 103 (6), 95 (17), 94 (32), 93 (55), 92 (17), 91 (57), 84 (9), 81 (12), 80 (6), 79 (57), 78 (8), 77 (33), 67 (14), 65 (10), 57 (5), 55 (13), 53 (11), 51 (7).
	**u13**	12.30	152 (8), 135 (12), 134 (100), 122 (6), 121 (54), 120 (6), 119

			(59), 117 (7), 110 (6), 109 (7), 106 (9), 105 (31), 103 (6), 95 (17), 94 (18), 93 (47), 92 (17), 91 (59), 84 (7), 81 (11), 80 (5), 79 (53), 78 (8), 77 (32), 67 (14), 65 (10), 55 (12), 53 (11), 51 (7).
	**u14**	13.50	150 (11), 135 (20), 132 (13), 122 (15), 121 (100), 119 (9), 117 (12), 115 (5), 109 (18), 108 (13), 107 (44), 106 (7), 105 (19), 95 (27), 94 (9), 93 (46), 92 (14), 91 (42), 89 (6), 83 (7), 82 (6), 81 (16), 80 (7), 79 (50), 77 (21), 73 (6), 71 (8), 69 (12), 67 (23), 65 (9), 59 (5), 58 (12), 57 (11), 55 (22), 53 (14), 51 (6).
(+)-Car-3-ene (**9**)	**u15**	9.06	138 (7), 137 (66), 123 (24), 121 (6), 119 (25), 110 (14), 109 (100), 108 (8), 107 (10), 105 (8), 96 (6), 95 (39), 94 (22), 93 (28), 92 (5), 91 (28), 83 (13), 82 (20), 81 (55), 80 (7), 79 (39), 77 (22), 71 (5), 69 (17), 68 (20), 67 (85), 65 (11), 55 (29), 54 (5), 53 (18), 51 (6).
	**u16**	11.05	119 (50), 117 (8), 115 (6), 110 (10), 109 (100), 108 (12), 107 (24), 106 (5), 105 (16), 103 (6), 97 (13), 96 (40), 95 (56), 94 (60), 93 (27), 92 (15), 91 (72), 84 (36), 83 (26), 82 (70), 81 (53), 80 (20), 79 (43), 78 (9), 77 (39), 70 (9), 69 (45), 68 (10), 67 (64), 66 (7), 65 (19), 59 (21), 57 (6), 56 (7), 55 (34), 54 (7), 53 (21), 51 (11).
	**u17**	11.91	134 (41), 121 (34), 120 (6), 119 (50), 117 (6), 109 (19), 108 (53), 107 (11), 106 (17), 105 (34), 103 (6), 97 (8), 95 (13), 94 (33), 93 (92), 92 (39), 91 (82), 84 (20), 83 (9), 82 (6), 81 (15), 80 (19), 79 (100), 78 (12), 77 (58), 69 (7), 67 (19), 65 (14), 57 (6), 55 (16), 53 (13), 51 (9).
1,8-Cineole (**10**)	**u18**	10.50	153 (100), 82 (89), 111 (85), 83 (83), 55 (59), 125 (43), 67 (38), 69 (21), 110 (20), 109 (17), 71 (14), 168 (14), 85 (13), 112 (13), 108 (13), 95 (13), 84 (12), 93 (12), 97 (11), 154 (10), 107 (10), 68 (10), 53 (9), 77 (8), 140 (7), 105 (7), 81 (6), 58 (6), 79 (5), 56 (5), 126 (5).
	**u19**	11.90	156 (10), 155 (100), 141 (6), 137 (27), 127 (31), 126 (6), 115 (9), 112 (8), 111 (22), 109 (23), 108 (39), 100 (9), 97 (9), 95 (11), 94 (16), 93 (87), 91 (6), 87 (30), 85 (53), 84 (25), 83 (20), 82 (10), 81 (10), 79 (14), 77 (7), 71 (29), 69 (40), 68 (10), 67 (14), 59 (40), 58 (29), 57 (31), 56 (24), 55 (22), 53 (9).
(+)-Fenchol (**11**)	**u20**	12.78	168 (11), 150 (5), 122 (5), 121 (19), 97 (20), 96 (11), 86 (6), 85 (100), 81 (7), 79 (6), 69 (5), 67 (5), 55 (7).
	**u21**	13.28	155 (25), 152 (20), 137 (29), 127 (5), 126 (20), 123 (18), 122 (9), 121 (76), 119 (7), 111 (40), 110 (10), 109 (45), 108 (23), 107 (7), 100 (9), 99 (14), 98 (11), 97 (48), 96 (20), 95 (24), 94 (8), 93 (24), 91 (12), 85 (41), 84 (10), 83 (31), 82 (21), 81 (100), 80 (83), 79 (27), 77 (13), 73 (9), 72 (49), 71 (33), 70 (12), 69 (35), 68 (14), 67 (26), 65 (7), 57 (31), 56 (22), 55 (38), 53 (16).
(-)-α-Pinene (**14**)	**u22**	11.70	168 (29), 153 (7), 138 (5), 137 (50), 135 (5), 125 (5), 110 (9), 109 (100), 108 (5), 107 (11), 95 (15), 94 (6), 93 (10), 91 (16), 85 (7), 81 (18), 79 (17), 77 (15), 69 (6), 67 (47), 65 (7), 55 (17), 53 (7).
	**u23**	12.15	150 (6), 135 (8), 127 (8), 111 (5), 110 (16), 109 (46), 108 (8), 107 (33), 105 (5), 99 (5), 98 (32), 97 (100), 96 (27), 95 (15), 93 (14), 92 (10), 91 (17), 85 (5), 84 (5), 83 (49), 82 (6), 81 (36), 80 (77), 79 (40), 77 (12), 71 (27), 70 (13), 69 (26), 68 (5), 67 (14), 65 (6), 57 (5), 55 (24), 53 (13), 51 (5).
	**u24**	14.20	221 (7), 208 (9), 163 (5), 151 (6), 150 (8), 147 (6), 141 (5),

			139 (8), 138 (7), 137 (31), 135 (51), 134 (9), 133 (5), 132 (6), 131 (11), 125 (11), 124 (7), 123 (6), 122 (12), 121 (47), 120 (9), 119 (19), 117 (20), 116 (5), 115 (12), 114 (6), 112 (8), 111 (7), 110 (9), 109 (15), 108 (18), 107 (83), 106 (12), 105 (27), 100 (7), 97 (15), 96 (19), 95 (100), 94 (16), 93 (35), 92 (41), 91 (61), 89 (12), 88 (6), 87 (16), 86 (5), 85 (21), 84 (5), 83 (18), 82 (20), 81 (23), 80 (18), 79 (77), 78 (13), 77 (44), 73 (11), 72 (19), 71 (10), 70 (9), 69 (52), 68 (11), 67 (28), 66 (14), 65 (23), 63 (8), 59 (30), 57 (21), 56 (6), 55 (47), 54 (5), 53 (23), 52 (7), 51 (11).
	**u25**	15.00	166 (22), 152 (5), 151 (51), 148 (9), 147 (7), 137 (5), 135 (27), 134 (5), 133 (28), 124 (10), 123 (30), 122 (5), 121 (42), 120 (11), 119 (12), 117 (5), 115 (6), 110 (7), 109 (30), 108 (11), 107 (60), 106 (18), 105 (87), 103 (10), 98 (8), 97 (8), 96 (46), 95 (100), 94 (9), 93 (48), 92 (17), 91 (79), 89 (6), 88 (8), 83 (15), 82 (9), 81 (20), 80 (14), 79 (66), 78 (21), 77 (53), 71 (5), 70 (49), 69 (25), 68 (7), 67 (48), 66 (12), 65 (24), 63 (8), 59 (8), 57 (5), 56 (6), 55 (63), 54 (6), 53 (27), 52 (8), 51 (16).
(+)-α-Pinene (**15**)	**u26**	13.50	152 (5), 151 (34), 148 (8), 147 (7), 138 (8), 137 (56), 135 (12), 134 (8), 133 (63), 131 (5), 124 (15), 123 (100), 122 (13), 121 (17), 120 (13), 119 (46), 117 (5), 115 (8), 111 (10), 110 (22), 109 (37), 108 (35), 107 (33), 106 (13), 105 (56), 104 (6), 103 (11), 98 (5), 97 (23), 96 (14), 95 (100), 94 (13), 93 (32), 92 (11), 91 (60), 89 (5), 85 (6), 83 (21), 82 (19), 81 (36), 80 (11), 79 (59), 78 (19), 77 (79), 72 (7), 70 (12), 69 (45), 68 (11), 67 (73), 66 (13), 65 (27), 63 (9), 59 (21), 57 (11), 56 (15), 55 (61), 54 (12), 53 (34), 52 (8), 51 (25).
	**u27**	15.00	166 (23), 152 (6), 151 (51), 148 (10), 147 (6), 138 (5), 135 (26), 133 (29), 124 (10), 123 (29), 122 (6), 121 (41), 120 (12), 119 (11), 117 (6), 115 (6), 110 (6), 109 (31), 108 (11), 107 (62), 106 (18), 105 (94), 103 (10), 98 (7), 97 (9), 96 (47), 95 (100), 94 (11), 93 (51), 92 (17), 91 (79), 88 (5), 83 (14), 82 (9), 81 (22), 80 (12), 79 (66), 78 (21), 77 (57), 71 (5), 70 (47), 69 (23), 68 (7), 67 (50), 66 (13), 65 (25), 63 (8), 59 (5), 56 (7), 55 (65), 54 (7), 53 (27), 52 (8), 51 (17).

Supplementary Table S17. Authentic standards used listed by retention time								
Product Name (number)	Supplier	Formula	Smiles	Pubchem CID	Retention time (min)	Quality against NIST		
						Match	R.Match	% Prob
(-)-*cis*-Limonene epoxide (**50**)	Sigma-Aldrich	$C_{10}H_{16}O$	CC(=C)[C@@H]1CC[C@@]2(C)OC2C1	6857487	9.04	953	966	40.5
(+)-*cis*-Limonene epoxide (**42**)	Sigma-Aldrich	$C_{10}H_{16}O$	C[C@]12C(O2)C[C@H](C(C)=C)CC1	6432653	9.04	941	951	35
(-)-*trans*-Limonene epoxide (**49**)	Sigma-Aldrich	$C_{10}H_{16}O$	C[C@@]12C(O2)C[C@H](C(C)=C)CC1	441245	9.15	949	957	49.9
(+)-*trans*-Limonene epoxide (**41**)	Sigma-Aldrich	$C_{10}H_{16}O$	C[C@@]12C(O2)C[C@H](C(C)=C)CC1	6432449	9.15	949	958	50.3
2,6-Dimethyl-3,7-octadien-2-ol (**20**)	Sigma-Aldrich	$C_{10}H_{18}O_2$	CC(CC=CC(C)(C)O)C=C	537103	9.3	904	905	79.1
(+)-Dihydrocarbone (-)[a]	Sigma-Aldrich	$C_{10}H_{16}O$	CC1CCC(CC1=O)C(=C)C	6432474	10.24	935	938	43.8
(S)-*cis*-Verbenol (**65**)	Sigma-Aldrich	$C_{10}H_{16}O$	CC1(C)[C@@H]2C[C@H]1[C@H](O)C=C2C	87839	10.52	955	960	30.9
(-)-*trans*-Pinocarveol (**60**)	Sigma-Aldrich	$C_{10}H_{16}O$	CC1([C@H]2C[C@@H]1C(=C)[C@@H](C2)O)C	1201530	10.52	943	964	45.3
(-)-Verbenone (**63**)	Sigma-Aldrich	$C_{10}H_{14}O$	CC1=CC(=O)C2CC1C2(C)C	29025	10.82	960	960	49.1
(-)-*trans*-Isopiperitenol (**47**)	In-house	$C_{10}H_{16}O$	CC1=CC(C(CC1)C(=C)C)O	439410	11.04	954	958	43.3
(-)-Myrtenol (**59**)	Sigma-Aldrich	$C_{10}H_{16}O$	CC1([C@H]2CC=C([C@@H]1C2)CO)C	88301	11.24	952	953	52.4
(-)-Myrtenal (**-**)	Sigma-Aldrich	$C_{10}H_{14}O$	CC1([C@H]2CC=C([C@@H]1C2)C=O)C	1201529	11.36	938	941	52.1
di-pentene-dioxide (**-**)	Sigma-Aldrich	$C_{10}H_{16}O_2$	CC12CCC(CC1O2)C3(CO3)C	232703	11.4	893	894	71.2
(-)-*cis*-Carveol (**45**)	Sigma-Aldrich	$C_{10}H_{16}O$	CC1=CC[C@H](C[C@H]1O)C(=C)C	330573	11.42	961	961	76.9
di-pentene-dioxide (**-**)	Sigma-Aldrich	$C_{10}H_{16}O_3$	CC12CCC(CC1O2)C3(CO3)C	232703	11.45	906	906	74.7
p-cymen-8-ol (**29**)	Sigma-Aldrich	$C_{10}H_{14}O$	CC1=CC=C(C=C1)C(C)(C)O	14529	11.46	936	936	70.3
di-pentene-dioxide (**-**)	Sigma-Aldrich	$C_{10}H_{16}O_4$	CC12CCC(CC1O2)C3(CO3)C	232703	11.48	884	884	68.7
(-)-*trans*-Carveol (**-**)	Sigma-Aldrich	$C_{10}H_{16}O$	CC(=C)[C@@H]1CC=C(C)[C@@H](O)C1	94221	11.56	962	962	70
cis-Myrtanol (**-**)	Sigma-Aldrich	$C_{10}H_{18}O_2$	CC1([C@@H]2CC[C@@H]([C@H]1C2)CO)C	117419	11.62	944	946	39.4
3-Caren-10-ol (**41**)	Chem-Space	$C_{10}H_{16}O$	OCC(CC12)=CCC2C1(C)C	9877427	11.93	-	-	-
(1R,2R,5R)-(+)-2-Hydroxy-3-pinanone (**-**)	Sigma-Aldrich	$C_{10}H_{16}O_2$	CC1([C@@H]2C[C@H]1C(C(=O)C2)(C)O)C	12760081	11.99	938	944	94.5

(1S,2S,5S)-(+)-2-Hydroxy-3-pinanone (-)	Sigma-Aldrich	$C_{10}H_{16}O_3$	C[C@@]1([C@H]2C[C@H](C2(C)C)CC1=O)O	11126668	11.99	944	952	94.5
(-)-Perillyl alcohol (**44**)	Sigma-Aldrich	$C_{10}H_{16}O$	CC(=C)C1CCC(=CC1)CO	10819	12.17	933	941	49.5
p-Cymen-7-ol (**27**)	Sigma-Aldrich	$C_{10}H_{14}O$	CC(C)C1=CC=C(C=C1)CO	325	12.55	942	942	75.3
Thymol (**26**)	Sigma-Aldrich	$C_{10}H_{14}O$	CC1=CC(=C(C=C1)C(C)C)O	6989	12.82	945	945	58.7
Carvacrol (**25**)	Sigma-Aldrich	$C_{10}H_{14}O$	CC1=C(C=C(C=C1)C(C)C)O	10364	12.94	937	937	35.2
Sobrerol (**33**)	Sigma-Aldrich	$C_{10}H_{18}O_2$	CC1=CCC(CC1O)C(C)(C)O	91463	13.34	920	923	69.7
7-hydroxy-terpineol (**32**)	Chem-Space	$C_{10}H_{18}O_2$	CC(C)(O)C1CCC(CO)=CC1	110662	14.13	-	-	-

[a](-): no number was assigned.

Supplementary Table S18. Product identification against NIST MS Library and/or authentic standards										
**Substrate**	**Retention time (min)**	**Identified product**	**Formula**	**Smiles**	**Pubchem CID**	**Empirical retention index**	**Match parameters on NIST Library**			**ID Level**
							**Match**	**R. Match**	**% Prob**	
(*R*/*S*)-Linalool (**1**)	9.28	*trans*-Linalool oxide (**18**)	$C_{10}H_{18}O_2$	C[C@]1(CC[C@H](O1)C(C)(C)O)C=C	6432254	1621	961	961	42.3	2
	9.76	Dihydrolinalool (**17**)	$C_{10}H_{20}O$	CCC(C)(CCC=C(C)C)O	86749	1546	870	922	85.4	2
	10.96	(*trans*)-Pyranoid linalool oxide	$C_{10}H_{18}O_2$	CC1(C(CCC(O1)(C)C=C)O)C	6427788	1764	896	915	73.5	2
	11.07	(*trans*)-Pyranoid linalool oxide	$C_{10}H_{18}O_2$	CC1(C(CCC(O1)(C)C=C)O)C	6427788	1859	911	913	62.8	2
	13.29	8-Hydroxylinalool (**16**)	$C_{10}H_{16}O$	CC(=CCCC(C)(C=C)O)CO	5280678	2000	954	955	86.8	2
(*E*/*Z*)-β-Ocimene (**2**)	9.24	(*Z*)-Myroxide (**22**)	$C_{10}H_{16}O$	C/C(=C/CC1C(O1)(C)C)/C=C	94648	1582	941	948	76.7	2
	9.4	(*E*)-Myroxide (**21**)	$C_{10}H_{16}O$	C\C(C=C)=C/CC1OC1(C)C	94648	1884	919	922	54.4	2
	11.32	(3E,5E)-2,6-Dimethyl-3,5,7-octatrien-2-ol (**20**)	$C_{10}H_{16}O$	CC(=CC=CC(C)(C)O)C=C	5363695	1817	889	895	37.5	2
Geraniol (**3**)	13.41	Geranic acid (**24**)	$C_{10}H_{16}O_2$	CC(=CCCC(=CC(=O)O)C)C	5275520	2000	922	947	49.8	2
	14.6	8-hydroxygeraniol (**23**)	$C_{10}H_{18}O_2$	CC(=CCO)CCC=C(C)CO	5363397	2000	969	976	89	2
(*R*)-Limonene (**7**)	9.05	*cis*-(-)-1,2-limonene epoxide (**42**)	$C_{10}H_{16}O$	C[C@]12C(O2)C[C@H](C(C)=C)CC1	6857487	1467	947	956	38.8	1
	9.16	(+)-*trans*-Limonene 1,2-epoxide (**41**)	$C_{10}H_{16}O$	C[C@@]12C(O2)C[C@H](C(C)=C)CC1	441245	1514	941	950	54.6	1
	10.24	D-Dihydrocarvone	$C_{10}H_{16}O$	CC1CCC(CC1=O)C(=C)C	24473	1603	808	837	38.4	1

	10.25	*trans*-d-hydrocarvone	$C_{10}H_{16}O$	C[C@H]1CC[C@@H](CC1=O)C(C)C	6432474	1606	951	953	58.2	2
	11.03	(-)-*cis*-Isopiperitenol (**40**)	$C_{10}H_{16}O$	CC1=C[C@H]([C@H](CC1)C(=C)C)O	12311192	1810	956	956	64.3	2
	11.05	(-)-*trans*-Isopiperitenol (**39**)	$C_{10}H_{16}O$	CC1=C[C@@H]([C@H](CC1)C(=C)C)O	439410	1830	957	959	43.2	1
	11.22	Perillyl aldehyde (-)	$C_{10}H_{14}O$	CC(=C)C1CCC(=CC1)C=O	16441	2751	944	945	65.1	2
	11.57	*trans*-Carveol (**37**)	$C_{10}H_{16}O$	CC1=CC[C@@H](C[C@H]1O)C(=C)C	443178	2003	932	932	48.7	1
	12.19	Perillyl alcohol (**36**)	$C_{10}H_{16}O$	CC(=C)C1CCC(=CC1)CO	10819	2000	938	941	48.2	1
	13.18	Limonene-1,2-diol (**35**)	$C_{10}H_{18}O_2$	CC(=C)C1CCC(C(C1)O)(C)O	94217	2000	910	923	83.7	2
(*S*)-Limonene (**8**)	9.05	*cis*-(-)-1,2-limonene epoxide (**50**)	$C_{10}H_{16}O$	CC(=C)[C@@H]1CC[C@@]2(C(C1)O2)C	6857487	1467	939	939	33.5	1
	9.16	*trans*-Limonene epoxide (**49**)	$C_{10}H_{16}O$	CC(=C)[C@@H]1CC[C@]2([C@@H](C1)O2)C	6432449	1514	958	968	49.6	1
	11.03	(-)-*cis*-Isopiperitenol (**48**)	$C_{10}H_{16}O$	CC1=C[C@H]([C@H](CC1)C(=C)C)O	12311192	1810	945	945	65.3	2
	11.05	(-)-*trans*-Isopiperitenol (**47**)	$C_{10}H_{16}O$	CC1=C[C@@H]([C@H](CC1)C(=C)C)O	439410	1830	948	948	38.4	1
	11.22	Perillyl aldehyde (-)	$C_{10}H_{14}O$	CC(=C)C1CCC(=CC1)C=O	16441	2751	939	943	63.1	2
	11.42	*cis*-Carveol (**46**)	$C_{10}H_{16}O$	CC(=C)[C@@H]1CC=C(C)[C@H](O)C1	330573	1854	880	884	43.1	1
	11.48	α-Limonene diepoxide (**45**)	$C_{10}H_{16}O$	CC12CCC(CC1O2)C3(CO3)C	232703	1891	895	922	65.3	2
	11.88	*trans*-Shisool (-)	$C_{10}H_{18}O$	CC(=C)C1CCC(CC1)CO	519954	1966	901	912	34.1	2

	12.18	Perillyl alcohol (**44**)	$C_{10}H_{16}O$	CC(=C)C1CCC(=CC1)CO	10819	2000	936	938	50.9	1
	13.18	Limonene-1,2-diol (**43**)	$C_{10}H_{18}O_2$	CC(=C)C1CCC(C(C1)O)(C)O	94217	2000	861	879	70.2	2
γ-Terpinene (**4**)	6.49	*p*-cymene	$C_{10}H_{14}$	CC1=CC=C(C=C1)C(C)C	7463	1368	923	923	14.7	2
	8.96	*p*-cymenene	$C_{10}H_{12}$	CC1=CC=C(C=C1)C(=C)C	62385	1445	938	941	19.7	2
	11.19	4-Isopropylbenzaldehyde (**31**)	$C_{10}H_{12}O$	CC(C)C1=CC=C(C=C1)C=O	326	2309	945	958	35.7	2
	11.28	1,4-*p*-Menthadien-7-al (**30**)	$C_{10}H_{14}O$	CC(C)C1=CCC(=CC1)C=O	6429112	1806	923	928	54.5	2
	11.47	*p*-Cymen-8-ol (**29**)	$C_{10}H_{14}O$	CC1=CC=C(C=C1)C(C)(C)O	14529	1888	958	958	73.3	1
	12.37	*p*-Mentha-1,4-dien-7-ol (**28**)	$C_{10}H_{16}O$	CC(C)C1=CCC(=CC1)CO	519966	2000	956	953	84.9	2
	12.55	*p*-Cymen-7-ol (**27**)	$C_{10}H_{14}O$	CC(C)C1=CC=C(C=C1)CO	325	2000	939	939	71.7	1
	12.83	Thymol (**26**)	$C_{10}H_{14}O$	CC1=CC(=C(C=C1)C(C)C)O	6989	2000	922	925	40.9	1
	12.95	Carvacrol (**25**)	$C_{10}H_{14}O$	CC1=C(C=C(C=C1)C(C)C)O	10364	2000	936	939	38.6	1
Terpinolene (**6**)	8.85	*p*-Mentha-1,5,8-triene (**-**)	$C_{10}H_{14}$	CC1=CCC(C=C1)C(=C)C	527424	1424	912	916	50.2	2
	12.53	*p*-Menthadien-7-ol (**34**)	$C_{10}H_{16}O$	CC(C)C1=CC=C(CC1)CO	556567	2000	854	858	54	2
α-Terpineol (**5**)	13.35	Sobrerol	$C_{10}H_{18}O_2$	CC1=CCC(CC1O)C(C)(C)O	91463	2000	849	856	50	1
	14.14	7-hydroxy-terpineol	$C_{10}H_{16}O_2$	CC(C)(O)C1CCC(CO)=CC1	110662	2000	-	-	-	1

1,8-cineole (**10**)	11.55	2-Hydroxycineole	$C_{10}H_{18}O_2$	CC1(C2CCC(O1)(C(C2)O)C)C	529885	1983	956	957	78.9	2
	11.78	3-exo-Hydroxy-1,8-cineole	$C_{10}H_{18}O_2$	CC1(C2CCC(O1)(CC2O)C)C	102149463	1920	888	900	95.6	2
(+)-Car-3-ene (**9**)	6.48	*p*-cymene	$C_{10}H_{14}$	CC1=CC=C(C=C1)C(C)C	7463	1368	914	919	16.7	1
	10.85	*p*-Mentha-1,5-dien-8-ol (**55**)	$C_{10}H_{16}O$	CC1=CCC(C=C1)C(C)(C)O	519323	1719	936	946	93.2	2
	10.89	*p*-Mentha-1,5-dien-8-ol (**55**)	$C_{10}H_{16}O$	CC1=CCC(C=C1)C(C)(C)O	519323	1734	923	928	90.9	2
	11.44	*m*-Cymen-8-ol (**53**)	$C_{10}H_{14}O$	CC1=CC(=CC=C1)C(C)(C)O	255195	1869	914	918	39.6	2
	11.87	Car-3-en-5-one (**52**)	$C_{10}H_{14}O$	CC1=CC(=O)C2C(C1)C2(C)C	13502875	1961	881	910	35.4	1
	11.93	3-Caren10-ol (**51**)	$C_{10}H_{16}O$	OCC(CC12)=CCC2C1(C)C	9877427	2026	-	-	-	1
	12.5	*p*-Cymen-7-ol (-)	$C_{10}H_{14}O$	CC(C)C1=CC=C(C=C1)CO	325	2000	862	866	54	2
Fenchol (**11**)	8.54	Fenchone (-)	$C_{10}H_{16}O$	CC1(C2CCC(C2)(C1=O)C)C	14525	2094	897	925	71.1	2
(+)-α-Pinene (**15**)	10.37	(1R)-(-)-Myrtenal (-)	$C_{10}H_{14}O$	CC1([C@H]2CC=C([C@@H]1C2)C=O)C	1201529	1639	876	876	43.4	2
	10.54	*cis*-Verbenol (**68**)	$C_{10}H_{16}O$	CC1=C[C@H](O)[C@H]2C[C@@H]1C2(C)C	87839	1746	965	965	39.9	2
	10.67	*tras*-verbenol (**67**)	$C_{10}H_{16}O$	CC1=C[C@@H](O)[C@H]2C[C@@H]1C2(C)C	89664	2038	954	962	54.5	1
	10.83	(-)-Verbenone (**66**)	$C_{10}H_{14}O$	CC1=CC(=O)C2CC1C2(C)C	29025	1714	961	961	45	2
	11.26	(-)-Myrtenol (**61**)	$C_{10}H_{16}O$	CC1(C2CC=C(C1C2)CO)C	88301	22569	932	934	48.5	1

	13.36	Sobrerol (**33**)	$C_{10}H_{18}O_2$	CC1=CCC(CC1O)C(C)(C)O	91463	2000	915	919	69	2
(-)-α-pinene (**14**)	10.53	*cis*-verbenol (**65**)	$C_{10}H_{16}O$	CC1=C[C@@H](O)[C@@H]2C[C@H]1C2(C)C	87839	1735	961	961	36.5	1
	10.66	*tras*-verbenol (**64**)	$C_{10}H_{16}O$	CC1(C)[C@@H]2C[C@H]1[C@@H](O)C=C2C	89664	1992	936	650	53.6	1
	10.83	(-)-Verbenone (**63**)	$C_{10}H_{14}O$	CC1[C@@H](C(C)=C2)C[C@H]1C2=O	29025	1714	959	960	45.2	2
	11.24	(-)-Myrtenol (**59**)	$C_{10}H_{16}O$	CC1(C)[C@H]2C[C@@H]1C(CO)=CC2	10582	4429	781	822	19	1
	12	Camphostene (-)	$C_{10}H_{16}O_2$	CC1(C)C2CC1C(C)(O)C(=O)C2	112005	2114	845	899	83.4	1
	13.36	Sobrerol (**33**)	$C_{10}H_{18}O_2$	CC1=CCC(CC1O)C(C)(C)O	91463	2000	923	926	70.7	1
(+)-β-Pinene (**13**)	10.53	*trans*-Pinocarveol (**62**)	$C_{10}H_{16}O$	CC1(C)C2CC1C(=C)[C@H](O)C2	1201530	1739	935	936	41.2	2
	11.24	Myrtenol (**61**)	$C_{10}H_{16}O$	CC1(C)[C@H]2C[C@@H]1C(CO)=CC2	10582	4869	929	939	49.2	2
	11.58	(-)-*cis*-Myrtanol (-)	$C_{10}H_{18}O$	CC1(C)C2CC1C(CO)CC2	11084102	2046	918	948	32.4	1
	12.18	Perillyl alcohol (**58**)	$C_{10}H_{16}O$	CC(=C)C1CCC(CO)=CC1	10819	2000	933	938	67.3	1
	12.53	*p*-Menthadien-7-ol (**34**)	$C_{10}H_{16}O$	CC(C)C1=CC=C(CC1)CO	556567	2000	849	851	51.4	2
	14.14	7-hydroxy-terpineol (**32**)	$C_{10}H_{16}O_2$	CC(C)(O)C1CCC(CO)=CC1	110662	2000	-	-	-	1
(-)-β-Pinene (**12**)	10.53	L-Pinocarveol (**60**)	$C_{10}H_{16}O$	CC1([C@H]2C[C@@H]1C(=C)[C@@H](C2)O)C	1201530	1634	941	962	47.6	
	11.24	Myrtenol (**59**)	$C_{10}H_{16}O$	CC1(C)[C@H]2C[C@@H]1C(CO)=CC2	10582	4462	902	903	47.8	1

	11.58	(-)-*cis*-Myrtanol (-)	$C_{10}H_{18}O$	CC1(C)C2CC1C(CO)CC2	11084102	2046	872	918	38.9	2
	12.18	Perillyl alcohol (**58**)	$C_{10}H_{16}O$	CC(=C)C1CCC(=CC1)CO	10819	2000	918	939	51.5	2
	12.53	*p*-Menthadien-7-ol (**34**)	$C_{10}H_{16}O$	CC(C)C1=CC=C(CC1)CO	556567	2000	856	864	52.5	1
	14.14	7-hydroxy-terpineol (**32**)	$C_{10}H_{16}O_2$	CC(C)(O)C1CCC(CO)=CC1	110662	2000	-	-	-	1

Product identification was done at different levels (ID Level): 1, identification against authentic standard; 2, identification against NIST Library

Supplementary Methods

Supplementary Table S19. Basic information about primers				
**Primer name**	**Application**	**Product Size (bp)**	**Template**	**Mutagenesis**
pJ6411_Fw	Inverse PCR	3749bp	pJBEI-6411	
pJ6411_Rv	Inverse PCR	3749bp	pJBEI-6411	
$P450_{cam}$+O_Fw	PCR & OE-PCR	1315bp & 2925bp	pETM11	
$P450_{cam}$+O_Rv	PCR & OE-PCR	1315bp & 2925bp	pETM11	
PdR_Fw	PCR & OE-PCR	1269bp & 2925bp	pET21b	
PdR_Rv	PCR & OE-PCR	1269bp & 2925bp	pET21b	
PdX+O_Fw	PCR & OE-PCR	318bp & 2925bp	pETM11	
PdX+O_Rv	PCR & OE-PCR	318bp & 2925bp	pETM11	
Fusion_Fw	OE-PCR	2925bp	P450 variant	
Fusion_Rv	OE-PCR	2925bp	P450 variant	
$P450_{BM3}$+O_Fw	PCR & OE-PCR	3206 bp & 6955 bp	pET15b	
$P450_{BM3}$+O_Rv	PCR & OE-PCR	30206 bp & 6955 bp	pET15b	
$P450_{cam}$-seq_Fw	Sequencing	nd	pJBEI-$P450_{cam}$	
$P450_{cam}$-seq_Rv	Sequencing	nd	pJBEI-$P450_{cam}$	
pBbseq_Rv	Sequencing	nd	pJBEI-$P450_{cam}$	
Y96F	Mutagenesis	6626 bp	Wt-$P450_{cam}$	Y96F-$P450_{cam}$
V447L_1	Mutagenesis	6626 bp	Y96F-$P450_{cam}$	Y96F-V247L-$P450_{cam}$
F87W	Mutagenesis	6626 bp	Y96F-V247L-$P450_{cam}$	F87W- Y96F-V247L-$P450_{cam}$
L244A	Mutagenesis	6626 bp	Y96F-$P450_{cam}$	Y96F- L244A-$P450_{cam}$
F87W	Mutagenesis	6626 bp	Y96F-L244A-$P450_{cam}$	F87W-Y96F-L244A-$P450_{cam}$
V247A	Mutagenesis	6626 bp	Y96F-L244A-$P450_{cam}$	Y96F-L244A-V247A-$P450_{cam}$
V247F	Mutagenesis	6626 bp	Y96F-L244A-$P450_{cam}$	Y96F-L244A-V247F-$P450_{cam}$
V247L_2	Mutagenesis	6626 bp	Y96F-L244A-$P450_{cam}$	Y96F-L244A-V247L-$P450_{cam}$
A328V	Mutagenesis	7075 bp	Wt-$P450_{BM3}$	A328V-$P450_{BM3}$
A264V	Mutagenesis	7075 bp	A328V-$P450_{BM3}$	A624V-A328V-$P450_{BM3}$
L437F	Mutagenesis	7075 bp	A264V-A328V-$P450_{BM3}$	A264V-A328V-L437F-$P450_{BM3}$
nd= not defined.				

Supplementary Table S20. Primers sequences.

Primer name	Sequence 5'-3'
pJ6411_Fw	TAAGGATCCAAACTCGAGTAAGG
pJ6411_Rv	CCTAGATCTTTTGAATTCCCAA
$P450_{cam}$+O_Fw	TTGGGAATTCAAAAGATCTAGGAGGATAAAGAAATGACCACCGAAACCATTCAGA
$P450_{cam}$+O_Rv	ACGTTATCATTGGCGTTCATTTCTTTATCCTCCTTTAAACTGCTTTGGTGGTTGC
PdR_Fw	ATGAACGCCAATGATAACGT
PdR_Rv	TTATGCGCTGCTCAGTTCTG
PdX+O_Fw	CAGAACTGAGCAGCGCATAAAGGAGGATAAAGAAATGAGCAAAGTTGTTTATGTTAGCC
PdX+O_Rv	CCTTACTCGAGTTTGGATCCTTATTACCACTGACGATCCGGAA
Fusion_Fw	TTGGGAATTCAAAAGATCTAGG
Fusion_Rv	CCTTACTCGAGTTTGGATCCTTA
$P450_{cam}$+O_Fw	TTGGGAATTCAAAAGATCTAGGAGGATAAAGAAATGACAATTAAAGAAATGCCTCAG
$P450_{cam}$+O_Rv	CCTTACTCGAGTTTGGATCCTTATTACCCAGCCCACACGTCT
$P450_{cam}$-seq_Fw	ACCGAAGATTATGCAGAACCG
$P450_{cam}$-seq_Rv	CTGTACCCGGTTTCTGACGA
pBbseq_Rv	CTCTAGTAGAGAGCGTTCAC
Y96F	GAAGCCGGTGAAGCCTTTGATTTTATTCCGACCAGC
V447L_1	GTGGTCTGCTGCTGCTTGGTGGTCTGGATACC
F87W	GCAGCGAATGTCCGTGGATTCCGCGTGAAGCCG
L244A	GCAAAACGTATGTGTGGTGCGCTGCTGGTTGGTGGTC
V247L_2	GGTGCGCTGCTGCTTGGTGGTCTGG
V247A	GGTGCGCTGCTGGCTGGTGGTCTGGATAC
V247F	GGTGCGCTGCTGTTTGGTGGTCTGGATAC
A328V	CGCTTATGGCCAACTGTTCCTGCGTTTTCCCTATATGC
A264V	GCTGCGCTTATGGCCAACTTTTCCTGCGTTTTCCCTATATGC
L437F	CTACGAGCTGGATATTAAAGAAACTTTCACGTTAAAACCTGAAG

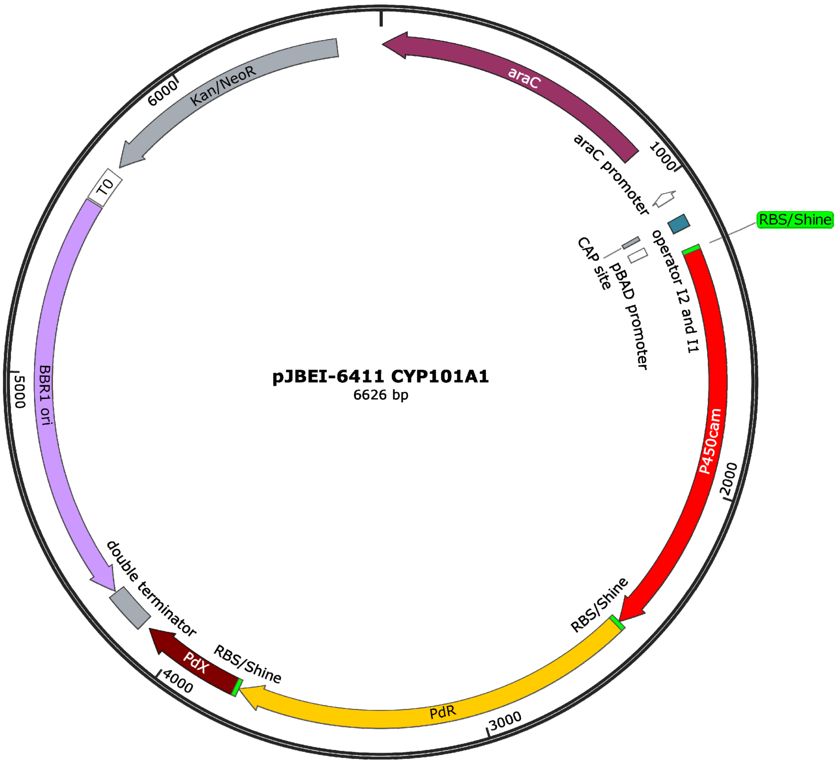

Supplementary Figure S16. pJBEI-6411 construct map.
